# Impact of gene annotation choice on the quantification of RNA-seq data

**DOI:** 10.1186/s12859-022-04644-8

**Published:** 2022-03-30

**Authors:** David Chisanga, Yang Liao, Wei Shi

**Affiliations:** 1 Olivia Newton-John Cancer Research Institute, Melbourne, Australia; 2 School of Cancer Medicine, La Trobe University, Melbourne, Australia; 3 Walter and Eliza Hall Institute of Medical Research, Melbourne, Australia; 4 Department of Medical Biology, The University of Melbourne, Melbourne, Australia; 5 School of Computing and Information Systems, The University of Melbourne, Melbourne, Australia

**Keywords:** RNA-seq, Gene expression quantification, Gene annotation, Normalization

## Abstract

**Background:**

RNA sequencing is currently the method of choice for genome-wide profiling of gene expression. A popular approach to quantify expression levels of genes from RNA-seq data is to map reads to a reference genome and then count mapped reads to each gene. Gene annotation data, which include chromosomal coordinates of exons for tens of thousands of genes, are required for this quantification process. There are several major sources of gene annotations that can be used for quantification, such as Ensembl and RefSeq databases. However, there is very little understanding of the effect that the choice of annotation has on the accuracy of gene expression quantification in an RNA-seq analysis.

**Results:**

In this paper, we present results from our comparison of Ensembl and RefSeq human annotations on their impact on gene expression quantification using a benchmark RNA-seq dataset generated by the SEQC consortium. We show that the use of RefSeq gene annotation models led to better quantification accuracy, based on the correlation with ground truths including expression data from >800 real-time PCR validated genes, known titration ratios of gene expression and microarray expression data. We also found that the recent expansion of the RefSeq annotation has led to a decrease in its annotation accuracy. Finally, we demonstrated that the RNA-seq quantification differences observed between different annotations were not affected by the use of different normalization methods.

**Conclusion:**

In conclusion, our study found that the use of the conservative RefSeq gene annotation yields better RNA-seq quantification results than the more comprehensive Ensembl annotation. We also found that, surprisingly, the recent expansion of the RefSeq database, which was primarily driven by the incorporation of sequencing data into the gene annotation process, resulted in a reduction in the accuracy of RNA-seq quantification.

**Supplementary Information:**

The online version contains supplementary material available at 10.1186/s12859-022-04644-8.

## Introduction

Gene expression profiling using RNA sequencing (RNA-seq) is a core activity in molecular biology. Comprehensive gene expression analysis in various settings is important for generating hypotheses for ongoing research, investigating drug-effects in biological or clinical settings and as a diagnostic tool. In this paper, we explore the fact that a popular approach in gene-level quantification from RNA-seq data involves mapping reads to a reference genome and then counting mapped reads associated with each gene [1–5]. The process of counting mapped reads to genes requires a database of known genes. A gene is only quantified if it or its components have genomic coordinates already defined with respect to the genome sequence in a process called annotation. For each genome annotation model, a different set of annotation techniques and information sources are used [6–9]. As such, these annotations vary in terms of comprehensiveness and accuracy of annotated genomic features. Annotations are an important component in an RNA-seq analysis as the results are dependent on what is known in the annotation database.

In this study, we focused on the Ensembl [6] and RefSeq [7] annotations, two of the mostly commonly used gene annotations for human and mouse. The annotation pipelines for generating RefSeq and Ensembl annotations both include an automated annotation process and a manual curation process. RefSeq and Ensembl use similar data sources in their automated annotation processes, including mRNA, EST, protein and RNA-seq data. However, it seems that Ensembl also uses the error-prone long-read RNA-seq data which are not utilized by RefSeq. Regarding manual curation, the curation performed for RefSeq seems to be more stringent than that performed for Ensembl. The RefSeq manual curation is based on both transcripts and literature whereas the Ensembl manual curation is predominantly based on the transcripts. RefSeq curators visualize transcript alignments and RNA-seq data to validate their gene models. They also use other data sources, which are not used by Ensembl curators to our knowledge, to seek further support for the annotated genes. For example, they utilize histone modification data to verify the existence of promoters. They also exploit CAGE (Cap Analysis of Gene Expression) data to validate the transcription start sites. The extensive and stringent manual curation performed in RefSeq is expected to lead to a higher gene annotation quality.

Despite the importance of gene annotations in RNA-seq data analysis, very little research has been conducted to examine how differences in annotations impact on gene expression quantification, which is crucial for downstream analyses such as discovery of differentially expressed genes and identification of perturbed pathways. Previous studies compared the effect of human genome annotations from popular databases including Ensembl and RefSeq on various aspects of RNA-seq analysis and they showed that the choice of annotations had an impact on gene-level quantification in the RNA-seq analysis [10, 11]. However, these studies are out of date as they were based on old annotations and they also lacked a reliable ground truth for assessing the impact of annotation.

The Ensembl and RefSeq annotation databases have undergone significant expansions over the years, thanks to the wide application of sequencing technologies and the massive amount of sequencing data that have been generated across the world. However, it is unclear whether the quality of gene annotations have been successfully maintained. A recent study suggested that gene annotations have become less accurate and lagging during this expansion [12]. This can be attributed to the errors from sequencing experiments, sequence analysis or automation in the annotation process. It is important to systematically assess the accuracy of the new gene annotations generated in recent years to ensure the popular annotation databases can continue to be utilized by the community for RNA-seq analysis.

Furthermore, the use of different annotations including Ensembl and RefSeq in different studies makes it difficult for researchers to reproduce the findings from such studies. There is a need to develop a comprehensive understanding of how these differences in annotations impact the gene-level expression quantification.

In this study, we compared three human gene annotations, including a recent Ensembl annotation, a recent RefSeq annotation and an old RefSeq annotation, to understand their impact on gene-level expression quantification in an RNA-seq data analysis pipeline. We were particularly interested in examining if the new annotations generated in recent years can help improve the accuracy of RNA-seq gene expression quantification. We used a benchmark RNA-seq dataset generated by the SEquencing Quality Control (SEQC/MAQC III) consortium for this evaluation. The accuracy of RNA-seq quantification results from using different annotations was assessed based on the correlation with ground truths including expression data from $>800$ real-time PCR validated genes, known genome-wide titration ratios of gene expression and microarray gene expression data. Finally, we investigated if any normalization method can mitigate the differences in quantification results caused by the annotation differences.

## Materials and methods

### SEQC/MAQC data

The RNA-seq data used for evaluation in this study are a benchmark dataset generated by the Sequencing Quality Control (SEQC) project [1], the third stage of the MicroArray Quality Control (MAQC) study [13, 14]. The SEQC dataset includes the Universal Human Reference RNA (UHRR) as sample A and the Human Brain Reference RNA (HBRR) as sample B. It also includes two other samples C and D, which are combination of A and B mixed in the ratios of 3:1 in C and 1:3 in D respectively. The samples were sequenced in four replicate paired-end libraries using an Illumina HiSeq 2000 sequencer at the Australian Genomics Research Facility (AGRF). Each library contains $\sim 20$ million 100 bp read pairs.

A TaqMan real-time polymerase chain reaction (RT-PCR) dataset with expression values measured for over 1000 genes, which was generated in the MAQC-I study [14], was used to validate the expression of the RNA-seq data in this study. The expression values were measured for both the UHRR and HBRR samples together with their respective combinations. Around 800–900 TaqMan RT-PCR genes, which had matching gene identifiers with expressed RNA-seq genes from different annotations, were included for assessing the accuracy of RNA-seq quantification. In addition, microarray data generated in the MAQC-I study with samples A to D hybridized to the Illumina Human-6 BeadChip microarrays were also used in the assessment. The TaqMan RT-PCR and Illumina microarray datasets are available as part of the Bioconductor package ‘seqc’ [15].

### Annotations used

Three human gene annotations were included in this study, including a recent Ensembl annotation, a recent RefSeq annotation and an old RefSeq annotation. All these annotations were generated based on the human reference genome GRCh38.

The Ensembl gene annotation used in this study was generated in April 2020. Its version number is 100. It was downloaded from ftp://ftp.ensembl.org/pub/release-100/gtf/homo_sapiens/Homo_sapiens.GRCh38.100.gtf.gz.

The recent RefSeq gene annotation used was released by the NCBI in August 2020. Its release number is 109.20200815 and it is part of the RefSeq release version 202. It was downloaded from the NCBI FTP site ftp://ftp.ncbi.nlm.nih.gov/refseq/H_sapiens/annotation/annotation_releases/109.20200815/GCF_000001405.39_GRCh38.p13/GCF_000001405.39_GRCh38.p13_genomic.gtf.gz. We refer this RefSeq annotation as ‘RefSeq-NCBI’ in this study.

The old RefSeq annotation included in this study was released by the NCBI in April 2015. It was released as part of the Patch 2 release of the GRCh38 genome build. This annotation has also been included in the popular RNA-seq quantification toolkit Rsubread [5] as the default annotation for quantifying human RNA-seq data. The RefSeq annotation in Rsubread is slightly different from the original one in that overlapping exons from the same gene were collapsed to form a single continuous exon. This modification however will not cause any difference to the gene-level RNA-seq quantification results as the set of exonic bases belonging to each gene remains the same. We refer this old RefSeq annotation as ‘RefSeq-Rsubread’.

The gene annotation data provided by Ensembl and RefSeq databases also contained biotype data for genes. These biotype data were used to assess the concordance and differences of gene biotypes between annotations. For genes included in the RefSeq-Rsubread annotation, their biotypes were predominantly annotated by using the biotype data included in the RefSeq-NCBI annotation. The biotypes of those RefSeq-Rsubread genes that were not included in RefSeq-NCBI annotation were manually annotated using the biotype data included in the old RefSeq annotation and also other resources.

When matching genes from different annotations, we converted the gene identifiers using the Bioconductor package ‘org.Hs.eg.db’ [16] and then compared them to find common genes between annotations.

### Mapping, quantification and normalization of RNA-seq data

Analysis of the RNA-seq data was performed using Bioconductor R packages Rsubread and limma [5, 17, 18]. The human reference genome (GRCh38) from GENCODE (version 34 downloaded from ftp://ftp.ebi.ac.uk/pub/databases/gencode/Gencode_human/release_34/GRCh38.primary_assembly.genome.fa.gz) was indexed using the buildindex function in Rsubread v2.2.6 [5]. Sequencing reads were then mapped to the reference genome using the align function in Rsubread [5, 19]. To successfully align a fragment (read pair), one end in the fragment must have at least three consensus votes (‘TH1 = 3’) from the ten extracted seeds (‘nsubreads = 10’) and the other end must have at least one consensus vote (‘TH2 = 1’). The fragment length computed from the mapping results of the two ends from the fragment was also required to be less than 600bp (‘maxFragLength = 600’). No more than three mismatches were allowed for the mapping of individual reads (‘maxMismatches = 3’). If only one end of a fragment can be mapped, it will be included in the mapping results as well. Multi-mapping fragments, which mapped to more than one location in the genome with equally best mapping quality, were reported with one best mapping location in the mapping output (‘unique = FALSE’ and ‘nBestLocations = 1’). See below for more details on the reporting of multi-mapping fragments.

The Ensembl, RefSeq-NCBI and RefSeq-Rsubread annotations were also provided to the align function for read mapping. For Ensembl and RefSeq-NCBI annotations, they were provided via the ‘annot.ext’ parameter. For RefSeq-Rsubread annotation, it was provided by specifying ‘annot.inbuilt = “hg38”’. The ‘useAnnotation’ parameter was also set to ‘TRUE’ when an annotation was provided to the align function.

The align function used the annotation data to try to break tie when reporting mapping results for multi-mapping fragments. If a multi-mapping fragment had only one mapping location found within an exon of a gene, this location would be selected as the final mapping location of the fragment. If multiple such locations were found, the location that first appeared in the index was chosen as the final mapping location. If no locations were found to overlap a gene, the location that first appeared in the index was chosen as the final mapping location.

Gene-level read counts were obtained with featureCounts [4, 5], a read count summarization function within the Rsubread package. The Ensembl, RefSeq-NCBI and RefSeq-Rsubread annotations were provided to featureCounts to generate read counts for genes included in these annotations respectively.

The gene-level read counts were transformed using the voom function in limma [17, 20] and then normalized using the library size [21], quantile [22] and trimmed mean of M-values (TMM) [23] methods, respectively, prior to performing further analysis. The library size normalization was performed by providing raw read counts to voom and then running voom with the ‘normalize.method’ parameter set to ‘none’. The quantile normalization was performed by providing raw read counts to voom and then running voom with the ‘normalize.method’ parameter set to ‘quantile’. For TMM normalization, we first calculated the TMM normalization factor for each library using the calcNormFactors method in edgeR [24]. Then we provided raw read counts and the TMM normalization factors to voom and ran it with the ‘normalize.method’ parameter set to ‘none’. The $\hbox {log}_2$CPM ($\hbox {log}_2$ counts per million) values, produced by the voom function for each gene in each library, were converted to $\hbox {log}_2$FPKM ($\hbox {log}_2$ fragments per kilo exonic bases per million mapped fragments) expression values for further analysis.

### Titration monotonicity

The RNA-seq data from the SEQC project have titration monotonicity built into them, such that a gene is considered to preserve titration monotonicity if the expression of the gene follows A $\ge$ C $\ge$ D $\ge$ B when its expression in sample A is greater than or equal to that in sample B, or follows A $\le$ C $\le$ D $\le$ B when its expression in sample A is less than or equal to that in sample B. To test if the titration monotonicity is preserved, Eq. (1) was used to compute the expected log2 fold-change for a gene in the comparison of C versus D given the log2 fold-change between A versus B.

1 $$\begin{aligned} E=log2\left( \frac{3\times 2^x+1}{2^x+3}\right) \end{aligned}$$

where *E* is the expected log2 fold-change for C versus D and *x* is the log2 fold-change for A versus B. Expression levels of genes in the replicates of the same sample were averaged before fold change of gene expression was calculated between samples.

### Validation

Gene expression data generated using TaqMan RT-PCR and Illumina’s BeadChip microarray were used to validate the gene-level quantification results from the RNA-seq analysis. Pearson correlation coefficients were computed to assess the concordance between the RNA-seq quantification data obtained from using different annotations and the gene expression data obtained from the RT-PCR and microarray experiments. The genome-wide built-in truth of titration monotonicity of gene expression in the RNA-seq data was also utilized to evaluate the quantification accuracy of RNA-seq data generated from using different annotations.

### Access to data and code

The data and analysis code used in this study can be accessed at the following URL: https://github.com/ShiLab-Bioinformatics/GeneAnnotation.

## Results

### Discrepancy between different gene annotations

The Ensembl and NCBI RefSeq annotations are among the most widely used gene annotations that have been utilized for RNA-seq gene expression quantification in the field. In this study, we examined a recent Ensembl annotation, a recent RefSeq annotation (‘RefSeq-NCBI’) and an older Refseq annotation (‘RefSeq-Rsubread’), to assess the impact of gene annotation choice on the accuracy of RNA-seq expression quantification. See Materials and Methods for more details of these annotations. The inclusion of an older RefSeq annotation allowed us to investigate the accuracy of new annotation data generated in recent years when the next-gen sequencing data have been used as a new data source for genome-wide annotation generation.

As RNA-seq gene-level expression quantification is typically performed for genes that contain exons [3–5], in this study we only focused on the genes that have annotated exons in each annotation. All the genes included in the downloaded Ensembl annotation contain at least one exon and they were all included in this study. The downloaded RefSeq-NCBI annotation contains 54,636 genes in total, however only 39,535 of them contain at least one exon. Genes that do not contain any annotated exons in this annotation were excluded from the study. Genes included in the RefSeq-Rsubread annotation all contain at least one exon and therefore they were all included in this study.

**Fig. 1:**
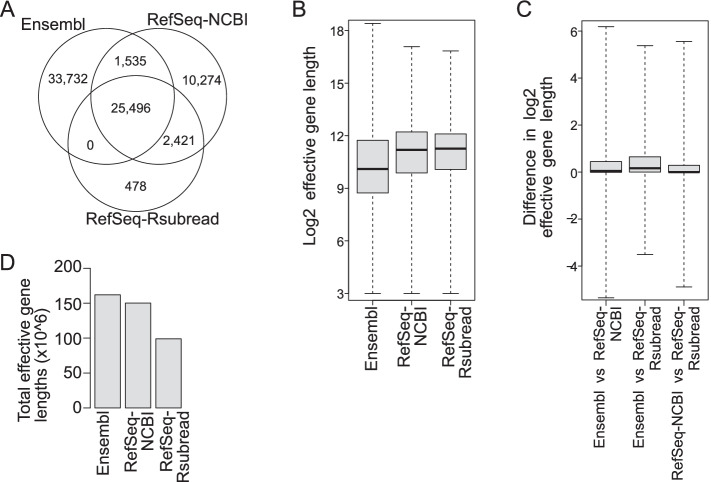
Concordance and differences between gene annotations. **A** Venn diagram showing genes that are common or unique in the Ensembl, RefSeq-NCBI and RefSeq-Rsubread annotations. **B** Boxplots showing the distribution of effective gene lengths ($log_2$ scale) in each annotation. **C** Boxplots showing the differences in effective lengths of common genes between each pair of annotations. Only genes that have a one-to-one mapping between the two annotations being compared were included in the analysis. Values shown in the plots are the ratio of effective lengths of the same gene from two different annotations ($log_2$ scale). **D** The size of transcriptome calculated from each annotation. Shown are the sum of effective gene lengths in each annotation

Figure 1A shows that the Ensembl annotation contains a lot more genes than the two RefSeq annotations. It is also worth noting that the RefSeq-NCBI annotation still has >12,000 genes absent from the Ensembl annotation even it contains less genes than Ensembl. Nearly 60% of the Ensembl genes are found to be absent from both of the two RefSeq annotations. In total, 25,496 common genes are found between the three annotations. Most of the genes included in the RefSeq-Rsubread annotation can be found in the RefSeq-NCBI or Ensembl annotations.

We also broke down genes according to their biotype and examined the overlap of genes between annotations for each biotype that is present in all annotations. Additional file 1: Fig. S1 shows that the three annotations are highly concordant in the categories of protein-coding genes and microRNAs. However, there are large number of pseudogenes and long non-coding RNAs (lncRNAs) found to be unique in both Ensembl and RefSeq annotations. Other non-coding RNAs, including small nucleolar RNAs, small nuclear RNAs, ribosomal RNAs and miscellaneous RNAs, also exhibited significant differences between Ensembl and RefSeq annotations. For the genes that are present in RefSeq-NCBI or RefSeq-Rsubread annotations but not in Ensembl annotation (13,173 genes), the majority of them are lncRNAs (Additional file 1: Fig. S2). For the genes that are unique to Ensembl (33,732 genes), most of them are lncRNAs and pseudogenes (Additional file 1: Fig. S3).

We then examined the effective gene lengths in each annotation. The effective length of a gene is the total number of unique bases included in all the exons belonging to the gene. Figure 1B shows the distributions of effective lengths of genes in the three annotations. Around half of the Ensembl genes have an effective length less than 1000 bases, whereas in the two RefSeq annotations only $\sim 25\%$ of the genes are shorter than 1,000 bases in length. The median effective gene lengths in RefSeq-NCBI and RefSeq-Rsubread are $\sim 3000$ bases, which is much larger than that in Ensembl ($\sim 1000$ bases). Although the Ensembl annotation contains a lot more genes than the two RefSeq annotations, it also contains a much higher percentage of short genes.

We further performed gene-wise comparison of effective gene lengths using common genes between each pair of annotations. Although every annotation contains both longer and shorter genes in comparison to the corresponding genes from other annotations, the Ensembl genes were found to have a larger effective length than genes from the two RefSeq annotations overall (Fig. 1C). This is in contrast to the higher proportion of short genes observed in the Ensembl annotation (Fig. 1B), which indicates that the Ensembl genes that are also present in RefSeq-NCBI or RefSeq-Rsubread annotations tend to be longer than those Ensembl genes that can only be found in the Ensembl annotation. Although at least half of the genes were found to have a less than 2-fold (1-fold at $log_2$ scale) length difference between annotations (Fig. 1C), the length differences could be as high as more than 64-folds (6-folds at $log_2$ scale). The RefSeq-NCBI genes seem to be slightly longer than the corresponding RefSeq-Rsubread genes overall. Ensembl and RefSeq-Rsubread were found to be the least concordant annotations among the three annotations being compared.

Lastly, we compared the size of the transcriptome represented by each annotation. The transcriptome size of an annotation is computed as the sum of effective gene lengths from all the genes included in that annotation, which also represents the total number of exonic bases that were annotated in an annotation. Figure 1D shows that the Ensembl annotation has a larger transcriptome size than both RefSeq-NCBI and RefSeq-Rsubread annotations. This is not surprising because the Ensembl annotation contains more genes and also Ensembl genes common to other annotations are longer in general. RefSeq-Rsubread has a much smaller transcriptome size than RefSeq-NCBI, indicating a significant expansion of the RefSeq-NCBI annotation in the past five years. However, it is important to note that the RefSeq-Rsubread annotation is not a subset of the RefSeq-NCBI annotation, as demonstrated by the existence of RefSeq-RSubread genes that are absent in the RefSeq-NCBI annotation, the difference in gene length distribution and the length differences of the same genes between the two annotations (Fig. 1A–C). This indicates that not only were new genes added to the RefSeq annotation during the expansion, but existing genes have been modified.

It is against this background that we sought to understand how these differences in the annotations impact on the overall gene-level quantification results.

### Fragments counted to annotated genes

We used a benchmark RNA-seq dataset generated by the SEQC project [1] to evaluate the impact of gene annotation on the accuracy of RNA-seq expression quantification. This dataset contains paired-end 100bp read data generated for four samples including a Universal Human Reference RNA sample (sample A), a Human Brain Reference RNA sample (sample B), a mixture sample with 75%A and 25%B (sample C) and a mixture sample with 25%A and 75%B (sample D).

**Fig. 2:**
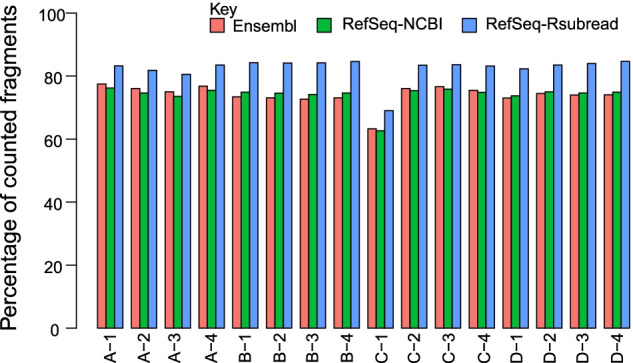
Barplots showing the percentage of fragments successfully assigned to genes in each annotation, out of all the fragments included in each library. The horizontal axis represents the sixteen SEQC RNA-seq libraries generated from the four samples ‘A’, ‘B’, ‘C’ and ‘D’. Each sample has four replicates that are numbered from 1 to 4

We mapped the RNA-seq reads to the human genome GRCh38 using the Subread aligner [5, 19], and then counted the number of mapped fragments (read pairs) to each gene in each annotation using the featureCounts program [4, 5]. FeatureCounts assigns a mapped fragment to a gene if the fragment overlaps any of the exons in the gene. Figure 2 shows that across all the 16 libraries, the RefSeq-Rsubread annotation constantly has substantially more fragments assigned to it than the Ensembl and RefSeq-NCBI annotations. This is surprising because RefSeq-Rsubread contains much less annotated genes and also has a significantly smaller transcriptome, compared to Ensembl and RefSeq-NCBI (Fig. 1A, D). We then performed a detailed investigation into the mapping and counting results to find out what enabled RefSeq-Rsubread to achieve a higher percentage of successfully assigned fragments.

Although gene annotations were utilized in mapping reads to the human reference genome, the use of different annotations was not found to affect the number of successfully aligned fragments for each library (Additional file 1: Fig. S4). We found that when assigning fragments to genes using the Ensembl or RefSeq-NCBI annotation, more fragments were unable to be assigned because they did not overlap any genes (ie. failed to overlap any exon included in any gene), despite there are more genes included in these annotations compared to the RefSeq-Rsubread annotation (Additional file 1: Fig. S5). This is particularly the case for the fragment assignment in the human brain reference samples. Additional file 1: Fig. S6 shows the read mapping results for a few selected genes that are only present in the RefSeq-Rsubread annotation. The annotations of these genes were generally well supported by the read mapping results.

We also found that the use of Ensembl and RefSeq-NCBI annotations led to more fragments being unassigned due to the assignment ambiguity, ie. a fragment overlaps more than one gene (Additional file 1: Figure S7). This should be because there are more genes that overlap with each other (ie. exons from different genes overlap with each other) in the Ensembl and RefSeq-NCBI annotations compared to the RefSeq-Rsubread annotation. Our investigation revealed that less gene overlapping in the RefSeq-Rsubread annotation and better compatibility of this annotation with the mapped fragments have led to more fragments being successfully counted for each library in this dataset. Given that both the Universal Human Reference and Human Brain Reference samples used in this study are known to contain a very high number of expressed genes and the RNA-seq data generated from these samples are expected to cover most of the human transcriptome, our analysis suggests that the RefSeq-Rsubread annotation is likely to contain more transcribed region in the genome than the other two annotations in general.

We would also like to note that multi-mapping fragments, which can be best mapped to more than one location in the genome, were also included in the read mapping results and subsequently assigned to the genes they overlap (see “Materials and Methods” for more details). Around 3–9% of total fragments in each library were found to be multi-mapping fragments (Additional file 1: Fig. S8). We performed an in-depth analysis to examine all the mapping locations found for them. 20–55% of multi-mapping fragments were found to map to two or more genes (ie. mapping to exons included in two or more genes) across the three annotations, however they only accounted for around 0.5–2.5% of total fragments in each library (Additional file 1: Fig. S9). In particular, when the NCBI-Rsubread annotation was used in the mapping, the percentage of such fragments was only $\sim$0.8% on average. As this is the only group of multi-mapping fragments that can cause assignment ambiguity, the very low percentage of these fragments means that they should not have much effect on the quantification results. Around 10–50% of multi-mapping fragments were found to map to one or more exons within the same gene, and 30–60% of them did not map to any exon in any gene, across the three annotations. These corresponded to around 0.5–4.5% and 1–3% of total fragments in each library, respectively (Additional file 1: Figs. S10 and S11).

As it is known that many multi-mapping fragments originate from pseudogenes, we particularly looked at the fraction of fragments that were assigned to these genes. As expected, significantly more fragments were assigned to pseudogenes in Ensembl ($\sim 6$%) than in RefSeq-NCBI and RefSeq-Rsubread ($<1$%)(Additional file 1: Fig. S12), due to much larger number of pseudogenes present in Ensembl.

### Intensity range of gene expression

**Fig. 3:**
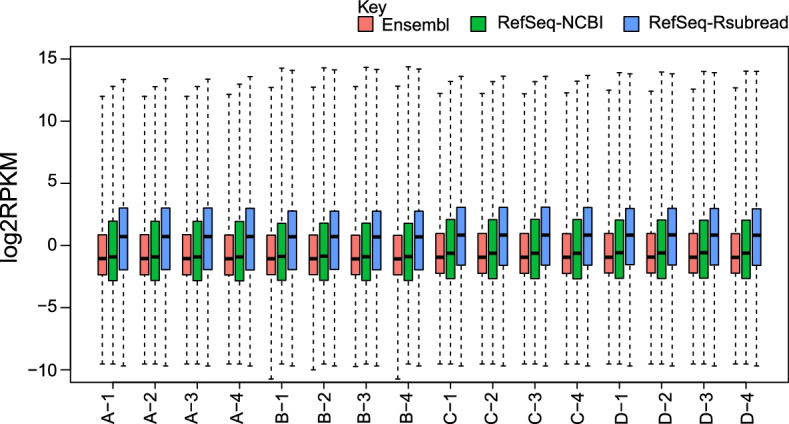
Boxplots comparing the intensity range of gene expression between the three annotations. All the genes from each annotation were included in the plots. Raw read counts of genes were transformed to $\hbox {log}_2$FPKM values. A prior count of 0.5 was added to raw counts to avoid log-transformation of zero

We examined if the gene annotation choice has an impact on the range of gene expression levels in the RNA-seq data. Raw gene counts of the SEQC data were converted to $\hbox {log}_2$FPKM ($\hbox {log}_2$ fragments per kilo exonic bases per million mapped fragments) values for all the genes included in each annotation. A prior count of 0.5 was added to the raw counts to avoid log-transformation of zero. Figure 3 shows that the two RefSeq annotations exhibit a desirable larger intensity range of gene expression than the Ensembl annotation, as shown by the larger boxes in the boxplots. It is surprising to see that the Ensembl genes have the smallest intensity ranges in all the libraries, give that the Ensembl annotation contains the largest number of genes in all the three annotations being examined. In addition to the large intensity range, the RefSeq-Rsubread genes were also found to have a markedly higher median expression level than genes in the RefSeq-NCBI and Ensembl annotations.

### Gene annotation discrepancy after expression filtering

As it is a common practice to filter out genes that are deemed as lowly expressed, or are completely absent in an RNA-seq data analysis [2], we also set out to assess the differences between alternative annotations after excluding such genes. We excluded those genes that failed to have at least 0.5 CPM (counts per million) in at least four libraries (each sample has four replicates) in the analysis of the SEQC dataset. The expression-filtered data were also used for comparing the accuracy of quantification from using alternative annotations presented in the following sections.

**Fig. 4:**
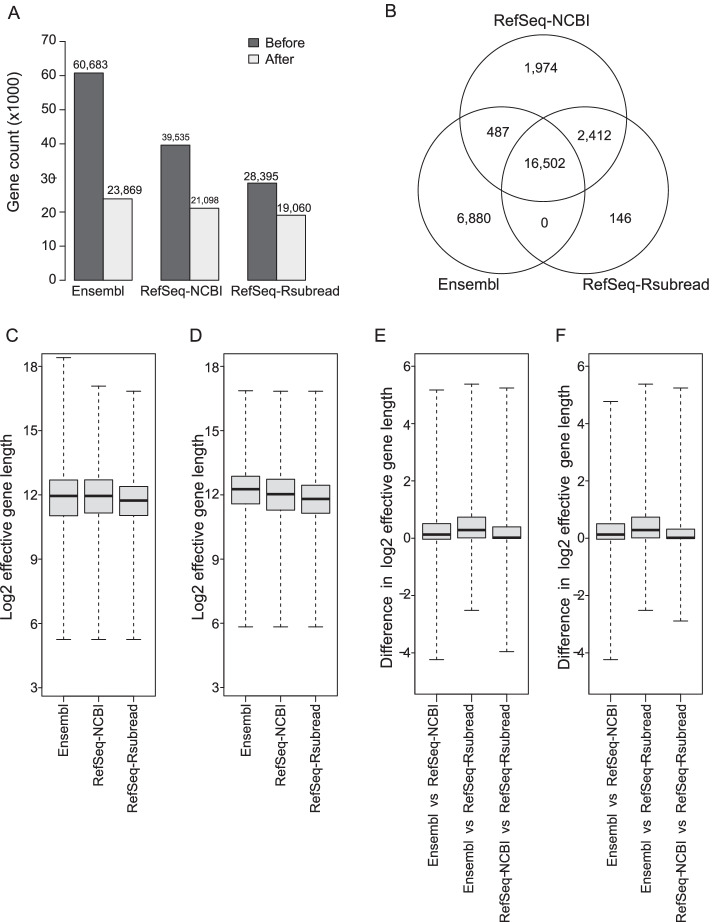
Concordance and differences between gene annotations after filtering for lowly expressed genes. **A** Bar plot showing the differences in the number of genes included in each annotation before and after filtering for lowly expressed genes. **B** Venn diagram comparing genes from different annotations after filtering for lowly expressed genes. Distributions of effective gene lengths after filtering are shown for all genes in each annotation **C** and for genes that are common between all three annotations (**D**). Distributions of differences of effective gene lengths between annotations after filtering are shown for common genes between each pair of annotations (**E**) and for genes that are common between all three annotations (**F**). Only genes that have a one-to-one mapping between the two annotations being compared were included in the analysis

The bar plot in Fig. 4A shows that Ensembl has significantly more genes (also higher proportion of genes) filtered out due to low or no expression, compared to RefSeq-NCBI and RefSeq-Rsubread. After expression filtering, the total numbers of remaining genes from the three annotations became more similar to each other. 16,472 genes were found to be common between the three annotations after filtering, accounting for 69%, 78% and 86% of the filtered genes in the Ensembl, RefSeq-NCBI and RefSeq-Rsubread annotations respectively (Fig. 4B). Almost all the filtered genes in the RefSeq-Rsubread annotation can be found in the other two annotations.

After expression filtering, the median effective gene length has increased to $\sim 4000$ bases for all annotations (Fig. 4C), meaning that a higher proportion of short genes were removed due to low expression in every annotation. The median effective length of Ensembl genes now became comparable to, or slightly higher than those in the two RefSeq annotations, indicating that the Ensembl annotation contained a higher proportion of lowly expressed short genes than the two RefSeq annotations. When comparing the effective lengths of genes common to all three annotations after filtering, the Ensembl genes were found to have the largest median effective length and the RefSeq-Rsubread genes have the smallest median effective length (Fig. 4D). This is not surprising because the Ensembl annotation is known to be more aggressive than the RefSeq annotations and RefSeq-Rsubread is an old annotation that has not been updated in the last five year.

The expression filtering did not seem to affect the distribution of differences of effective gene lengths between each pair of annotations (using genes common to each pair of annotations), with Ensembl and RefSeq-Rsubread remaining to be the least concordant annotations (Fig. 4E and 1C). Using genes common to all three annotations after filtering exhibited similar distributions of gene length differences between each pair of annotations compared to using genes common to each pair of annotations (Fig. 4F). Similar to before filtering, the gene-wise length comparison performed after filtering also showed that overall the Ensembl genes had the largest gene lengths and the RefSeq-Rsubread genes had the shortest gene lengths.

### Comparison of titration monotonicity preservation

To assess the impact of gene annotation choice on the accuracy of RNA-seq quantification result, we utilized as ground truth the inbuilt titration monotonicity in the SEQC data, the TaqMan RT-PCR data and the microarray data generated for the same samples, to evaluate which annotation gives rise to a better expression correlation of the RNA-seq quantification data with the truth.

**Fig. 5:**
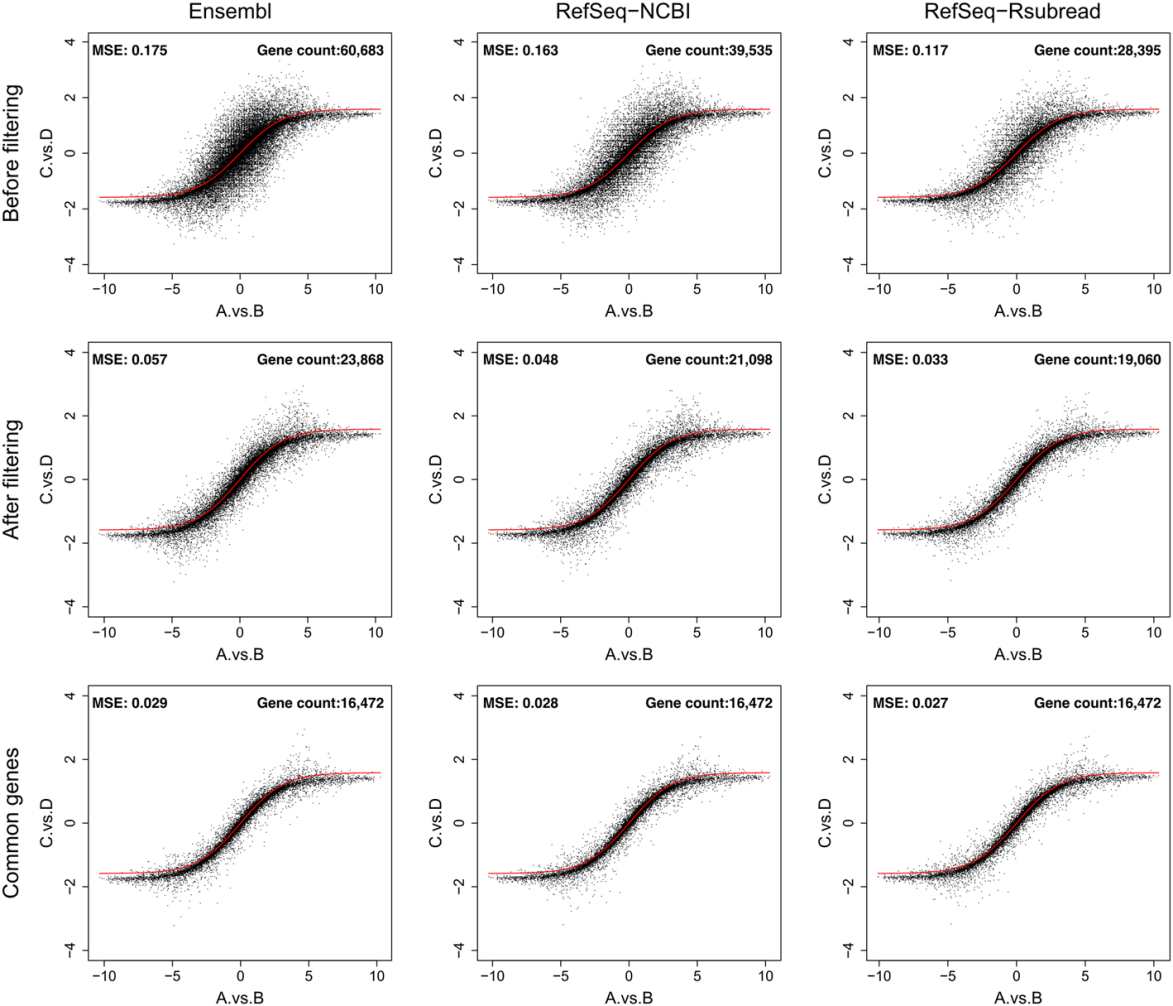
Titration monotonicity plots. The ability of Ensembl, RefSeq-NCBI and RefSeq-Rsubread to retain the titration monotonicity built into the SEQC RNA-seq data was measured using the Mean Squared Error (MSE) between the reference titration and the actual titration obtained from each annotation. The red curve in each plot represents the reference titration calculated from using Equation (1). Plots in the top row include all the genes available in each annotation. Plots in the middle row includes those genes that remained after filtering for lowly expressed genes, in each annotation. Plots in the bottom row includes genes that are common between the three annotations after the expression filtering was performed. In each plot, the horizontal axis represents the $\hbox {log}_2$ fold changes of gene expression between samples A and B and the vertical axis represents the $\hbox {log}_2$ fold changes of gene expression between samples C and D

In this section, we compared the ability of Ensembl and the two RefSeq annotations in retaining the inbuilt titration monotonicity in the RNA-seq dataset. In Fig. 5, the reference titration curve depicts the expected fold change that genes are expected to follow in sample C versus sample D based on the fold change in sample A versus sample B. This is computed using the Eq. (1) (see “Materials and methods”). We then calculated the Mean Squared Error (MSE) between the reference titration monotonicity and the titration monotonicity obtained from each annotation. A smaller MSE value means that the generated quantification data is closer to the truth. Figure 5 shows that the MSE computed for the RefSeq-Rsubread annotation is constantly lower than those computed for the Ensembl and RefSeq-NCBI annotations, regardless if filtering was applied or if only common genes were included for comparison. RefSeq-Rsubread was also found to yield comparable or lower MSE compared to the other two annotations when the data were TMM or quantile normalized (Additional file 1: Figs. S13 and S14), in addition to the library-size normalized data shown in Fig. 5. These results demonstrated that the use of RefSeq-Rsubread annotation led to better quantification accuracy for the RNA-seq data.

### Validation against TaqMan RT-PCR data

The TaqMan RT-PCR dataset generated in the MAQC study [13, 14] was used to validate the gene-level quantification results from the RNA-seq dataset. This dataset contains measured expression levels for $>1000$ genes in the four SEQC samples. The aim was to understand how well Ensembl and RefSeq annotated gene expression correlated with the TaqMan RT-PCR data.

The RNA-seq data generated from each annotation were filtered to remove lowly expressed genes before being compared to the RT-PCR data. Numbers of matched genes between the RT-PCR data and the RNA-seq data were 856, 901 and 901 for Ensembl, RefSeq-NCBI and RefSeq-Rsubread, respectively. 846 RT-PCR genes were found to be common to all the three annotations. The raw TaqMan RT-PCR data were $\hbox {log}_2$-transformed before comparing to the filtered RNA-seq data.

**Fig. 6:**
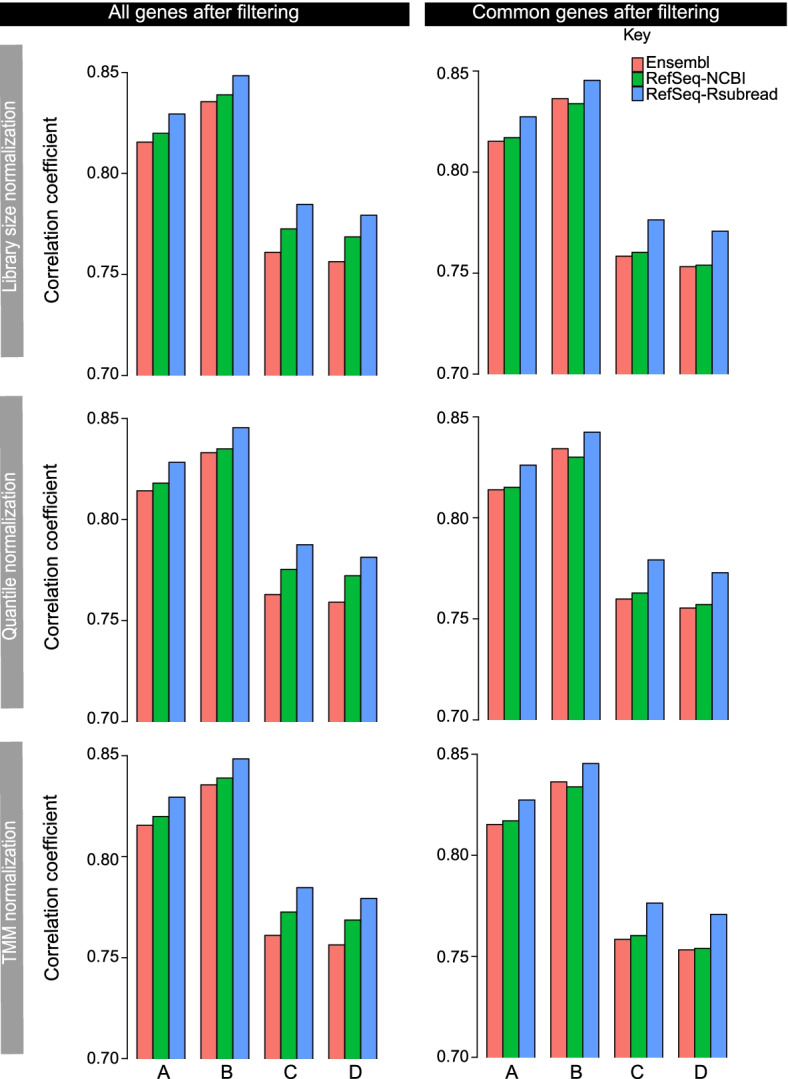
Validation of RNA-seq against TaqMan RT-PCR dataset. Shown are Pearson correlation coefficients computed from comparing RNA-seq data against RT-PCR data, using the RT-PCR genes matched with each individual annotation (left column) or matched with all three annotations (right column). The rows represent the different RNA-seq normalization methods used. Lowly expressed genes in the RNA-seq data were filtered out before the correlation analysis was performed

Pearson correlation analysis of the RNA-seq gene expression ($\hbox {log}_2$FPKM values) and RT-PCR gene expression ($\hbox {log}_2$ values) from using the RT-PCR genes matched with each individual annotation showed that the RefSeq-Rsubread annotation constantly yielded a higher correlation than the Ensembl and RefSeq-NCBI annotations, across all the samples and the three different normalization methods (left panel in Fig. 6). The Ensembl annotation was found to produce the worst correlation in all these comparisons. When using the RT-PCR genes matched with all three annotations for comparison, RefSeq-Rsubread was again found to yield the highest correlation (right panel in Fig. 6). Ensembl and RefSeq-NCBI were found to produce similar correlation coefficients. Taken together, results from this evaluation showed that the use of RefSeq-Rsubread annotation led to a better concordance in gene expression between the RNA-seq data and the RT-PCR data, compared to the use of Ensembl and RefSeq-NCBI annotations.

### Validation against microarray data

An Illumina BeadChip microarray dataset, which was generated by the MAQC-I project [14] for the same samples as in the RNA-seq data used in this study, was used to further validate the gene-level RNA-seq quantification results obtained from different annotations. The microarray dataset was background corrected and normalized using the ‘neqc’ function in the limma package [17, 25]. Microarray genes were then matched to the RNA-seq genes included in the filtered RNA-seq data. 14,405, 14,561 and 14,508 microarray genes were found to be matched with RNA-seq genes from Ensembl, RefSeq-NCBI and RefSeq-Rsubread annotations, respectively. 13,424 microarray genes were found to be present in all three annotations. For those microarray genes that contain more than one probe, a representative probe was selected for each of them. The representative probe selected for a gene had the highest mean expression value across the four samples among all the probes the gene has.

**Fig. 7:**
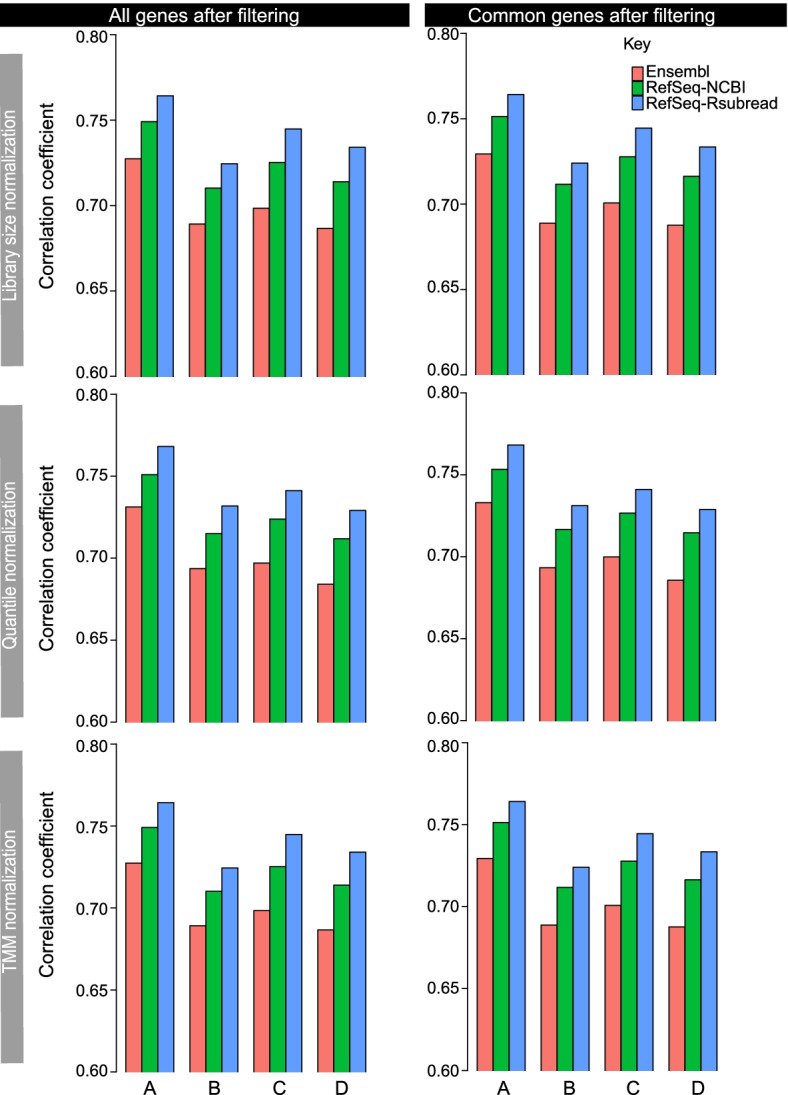
Validation of RNA-seq quantification results against microarray data. Shown are Pearson correlation coefficients computed from comparing RNA-seq data against Illumina BeadChip microarray data, using the microarray genes matched with each individual annotation (left column) or matched with all three annotations (right column). Rows in the plots represent the different RNA-seq normalization methods used. Lowly expressed genes in the RNA-seq data were filtered out before the correlation analysis was performed. For those microarray genes that include more than one probe, a representative probe was selected and used for this analysis

A Pearson correlation analysis was then performed between microarray data and RNA-seq data for each of the three annotations. Both RNA-seq and microarray data include $\hbox {log}_2$ expression values of genes. Figure 7 shows that the use of RefSeq-Rsubread annotation consistently yielded the highest correlation between RNA-seq and microarray data in all the comparisons, no matter which RNA-seq normalization method was used and if all or common matched genes were included in the evaluation. On the other hand, the use of the Ensembl annotation resulted in the worst correlation between RNA-seq data and microarray data in all the comparisons.

## Discussion

The RNA-seq technique is currently routinely used for genome-wide profiling of gene expression in the biomedical research field. The analysis of RNA-seq data relies on the accurate annotation of genes so that expression levels of genes can be accurately and reliably quantified. There are several major gene annotation sources that have been widely adopted in the field such as Ensembl and RefSeq annotations. The Ensembl and RefSeq annotations have been well maintained and under continuous development. In particular, new gene information collected from the next-generation sequencing technologies, such as RNA-seq, has been incorporated into the expansion of these annotations in recent years. However, differences between these annotations have raised concerns over the quality and reproducibility of RNA-seq data analyses. There are particularly concerns regarding the accuracy of new gene annotations generated from the use of next-generation sequencing technologies, which produced short reads with sequencing errors included (albeit the error rate is much lower now than some years ago). The short read length poses a challenging problem for generating accurate gene models due to the difficulty in assembling them into much longer gene transcripts. Long reads seem to be used for reconstructing transcripts in Ensembl, however the high error-rate associated with long reads is likely to result in errors in transcript reconstruction. Therefore, the inclusion of sequencing data into the gene annotation generation process poses a risk to the accuracy of gene models recently generated in major annotation databases.

To address these concerns, in this study we systematically assessed the differences in RNA-seq quantification results attributed to the gene annotation discrepancy. Annotations being evaluated in this study included recent Ensembl and NCBI RefSeq annotations and also an older version of the RefSeq annotation. We compared the recent and old RefSeq annotations to assess the quality of the new annotations that were added when the sequencing technology was utilized at NCBI for curating RefSeq gene annotations.

Although the Ensembl annotation contains significantly more genes than both the recent and old RefSeq annotations, it was also found to have a much higher proportion of short genes. Interestingly, we found that a much higher fraction of these short genes in Ensembl were filtered out due to low or no expression in the analysis of the SEQC RNA-seq dataset, compared to the short genes included in the two RefSeq annotations. The SEQC RNA-seq data is a widely used benchmark dataset including the Human Brain Reference RNA and Universal Human Reference RNA samples, in which a very large number of gene expressed making the entire human transcriptome well covered.

The use of the RefSeq-Rsubread annotation (the older version of the RefSeq annotation used in this study) has led to substantially more fragments being successfully counted to genes than the use of RefSeq-NCBI (the recent RefSeq annotation used in this study) or Ensembl annotations. A detailed investigation revealed that this was because (a) there are less overlapping between genes in the RefSeq-Rsubread annotation leading to less read assignment ambiguity and (b) the RefSeq-Rsubread annotation contains more genes that are compatible with mapped fragments, despite the transcriptome represented by this annotation is much smaller than those represented by the RefSeq-NCBI and Ensembl annotations. Moreover, the quantification data obtained from using RefSeq-Rsubread exhibited desirable larger intensity range and higher median expression level than the quantification data obtained from using the other two annotations.

The evaluation of quantification accuracy from using genome-wide titration monotonicity truth built in the RNA-seq data, the TaqMan RT-PCR data and the microarray data, showed that overall the RefSeq-NCBI annotation yielded better quantification results than the Ensembl annotation. This may not be surprising because the NCBI RefSeq annotation is a traditionally conservative annotation that is known to be highly accurate thanks to its high-quality curation process. However, we also found that the RefSeq-Rsubread annotation yielded more accurate quantification results than the RefSeq-NCBI annotation in almost all the comparisons, which is very surprising. We suspect that this might be due to the annotation errors such as the errors in reconstructing gene transcripts, arising from the use of short-read sequencing data recently adopted in the NCBI RefSeq annotation generation pipeline. It has been reported that the sequencing data, including RNA-seq data and epigenome sequencing data, started to be utilized by NCBI for curating RefSeq gene annotations in around 2013 [7, 26]. Between March 2015 and July 2020, the number of gene transcripts in the vertebrate mammalian organisms included in the RefSeq database increased significantly from 3.6 million to 7.8 million (https://www.ncbi.nlm.nih.gov/refseq/statistics/), a more than twofold increase in just around 5 years. The use of sequencing data for annotation generation should be a significant driver for this rapid expansion of the RefSeq database. It is known that some errors associated with the generation and analysis of sequencing data are difficult to correct, such as sample contamination, sequencing errors, read mapping errors and transcript assembly errors. When these errors were brought to the annotation process, they could result in incorrect gene annotations being generated and consequently led to less accurate quantification of the RNA-seq data.

We also tried to run the STAR aligner on the SEQC data to assess the impact of annotation choice on RNA-seq quantification accuracy. We found that the percentages of fragments successfully mapped by STAR in the sixteen libraries are similar to those from the Subread aligner (Additional file 1: Figs. S15 and S4). Around 5–9% of fragments were reported as multi-mapping fragments by STAR, which were modestly higher than those from Subread (Additional file 1: Figs. S16 and S8). However, the percentages of successfully assigned fragments from using STAR mapping results were found to be markedly lower than those from using Subread mapping results (Additional file 1: Figure S17 and Figure 2). In line with the accuracy comparison results from using Subread mapping results, the evaluations using STAR mapping results also showed that the RefSeq-Rsubread annotation yielded more accurate RNA-seq quantification results than Ensembl and RefSeq-NCBI annotations (Additional file 1: Figs. S18–S22). Interestingly, the use of Subread mapping results produced higher quantification accuracy than the use of STAR mapping results in almost all the comparisons (Figures 5-7 and Additional file 1: Fig. S13, S14, S18–S22).

The presence of multi-mapping fragments that mapped to two or more genes (ie. mapped to exons included in two or more genes) caused assignment ambiguity. The Subread+featureCounts approach for dealing with this is to assign them to the gene that first appears in the reference genome. This is not an accurate approach, but it did not seem to have much effect on the overall quantification accuracy due to the low percentage of such fragments in the data. We found that such fragments only accounted for $<2.5$% of total fragments in each library in the SEQC data across the three annotations, and accounted for $<1$% of total fragments when RefSeq-Rsubread annotation was used (Additional file 1: Fig. S9). Despite the small percentage of such fragments, the Subread+featureCounts approach can be improved by using a probabilistic algorithm, which considers the fragment abundance of relevant genes, for the assignment of these fragments.

Transcriptome-based RNA-seq quantification methods, such as RSEM [27] and Kallisto [28], employ statistical approaches to determine the assignment of reads to transcripts, including the assignment of multi-mapping reads that map to more than one transcript. We also ran RSEM and Kallisto on the SEQC dataset used in this study to evaluate how the choice of gene annotation could affect the quantification results from these methods. Both RSEM and Kallisto identified $\sim 40{-}70$% of fragments as multi-mapping fragments that mapped to two or more transcripts (Additional file 1: Figs. S23 and S24). They reported more multi-mapping fragments when Ensembl annotation is used, compared to the use of the two RefSeq annotations. Least number of multi-mapping fragments were identified when the RefSeq-Rsubread annotation was used. RSEM and Kallisto also assigned more fragments to Ensembl transcripts than to RefSeq-NCBI or RefSeq-Rsubread transcripts (Additional file 1: Figs. S25 and S26). This is different from the read assignment results from Subread+featureCounts where more fragments were assigned to RefSeq-Rsubread genes (Fig. 2). Notably, the average assignment percentage from the use of RefSeq-Rsubread annotation with Subread+featureCounts across libraries (82.5%) is comparable with those from the use of Ensembl annotation with RSEM (79.5%) and Kallisto (83.7%).

Lastly, we assessed the quantification accuracy of RSEM and Kallisto based on the preservation of titration monotonicity and correlation with RT-PCR and microarray data. We ran the Sleuth program [29] to summarise the transcript counts generated by Kallisto into gene-level counts, as Kallisto does not generate gene-level counts. RSEM can directly generate gene-level counts in addition to its transcript-level counts. Our evaluation results from using gene-level counts showed that, in line with the results from Subread+featureCounts, the use of RefSeq-Rsubread annotation also yielded better overall accuracy than the use of the Ensembl and RefSeq-NCBI annotations for RSEM and Kallisto/Sleuth (Additional file 1: Figures S27-S36). This further strengthened the conclusion from this study. Interestingly, Subread+featureCounts was found to achieve an overall higher accuracy than RSEM and Kallisto/Sleuth across all annotations (Figs. 5, 6, 7 and Additional file 1: Figs. S27–S36).

## Conclusion

In conclusion, our findings from this study revealed that the NCBI RefSeq human gene annotations outperformed the Ensembl human gene annotation in the quantification of RNA-seq data. However, we also raised concerns over the recent changes made to the RefSeq database due to the use of sequencing data in the annotation generation process. These changes need to be reviewed and validated so as to ensure the RefSeq database continues to be a reliable and high-quality gene annotation resource for the research community. Similarly, such review should be conducted for other gene annotation databases as well.

The research findings from this study also have an implication for the quantification of RNA-seq data generated by the recently emerged single-cell sequencing technologies. Same as the quantification of bulk RNA-seq data, an accurate gene annotation is also required for quantifying single-cell RNA-seq data. It is therefore important to understand if and how the annotation choice impacts the quantification accuracy of the single-cell RNA-seq data as well.

## Supplementary Information

**Additional file 1: Figures S1–S36.**

## Data Availability

The data and analysis code used in this study can be accessed at the following URL: https://github.com/ShiLab-Bioinformatics/GeneAnnotation.

## Abbreviations

RNA-seq: RNA sequencing
SEQC: SEquencing Quality Control
UHRR: Universal Human Reference RNA
HBRR: Human Brain Reference RNA
FPKM: fragments per kilo exonic bases per million mapped fragments
NCBI: National Center for Biotechnology Information
