# Supplementary material for: Impact of gene annotation choice on the quantification of RNA-seq data
Source: BMC Bioinformatics. 2022 Mar 30;23:107. doi: 10.1186/s12859-022-04644-8 (PMC8969366; doi:10.1186/s12859-022-04644-8)
Supplement: Supplementary file 1 — Additional file 1: Figures S1–S36. [file 12859_2022_4644_MOESM1_ESM.pdf]

Impact of gene annotation choice on the quantification of RNA-seq data – Supplementary Materials

David Chisanga[1,2,3,4], Yang Liao[1,2,3,4], and Wei Shi[1,2,3,5]

[1]Olivia Newton-John Cancer Research Institute, Heidelberg, Victoria, 3084, Australia
[2]School of Cancer Medicine, La Trobe University, Bundoora, Victoria, 3083, Australia,
[3]Walter and Eliza Hall Institute of Medical Research, Parkville, Victoria, 3052, Australia,
[4]Department of Medical Biology, The University of Melbourne, Parkville, Victoria, 3010, Australia,
[5]School of Computing and Information Systems, The University of Melbourne, Parkville, Victoria, 3010, Australia

This document includes Supplementary Figures S1-S36.

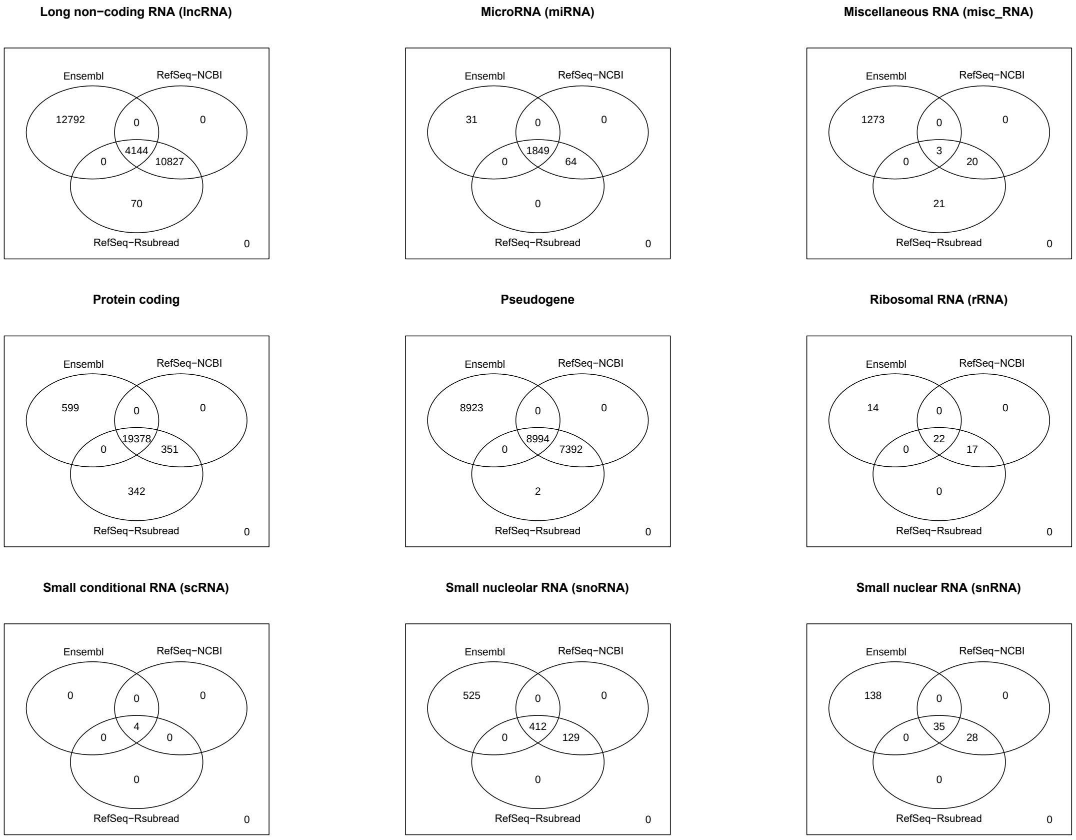

Figure S1. Venn diagrams showing numbers of genes that are common or unique between Ensembl, RefSeq-NCBI and RefSeq-Rsubread annotations for different biotypes. The biotypes shown here include all the common biotypes that can be found for the three annotations.

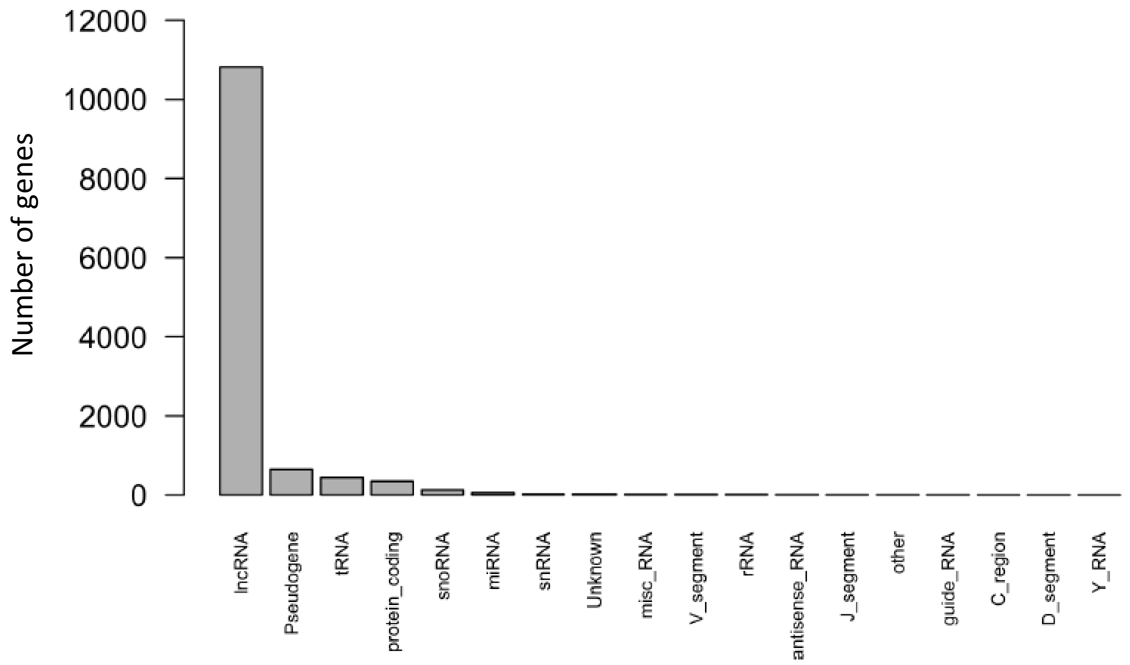

Figure S2. Barplot showing biotypes of genes that are present in RefSeq-NCBI or RefSeq-Rsubread annotations, but not in Ensembl annotation. The vertical axis shows the number of genes in each biotype.

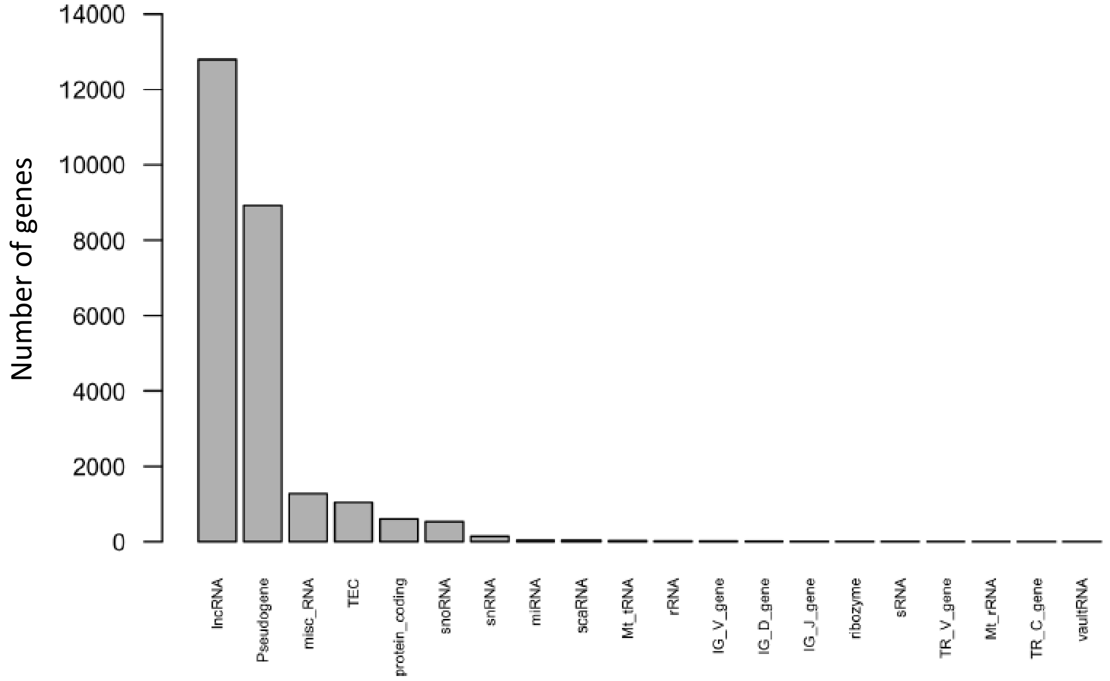

Figure S3. Barplot showing biotypes of genes that are present in Ensembl annotation, but not in RefSeq-NCBI or RefSeq-Rsubread annotations. The vertical axis shows the number of genes in each biotype.

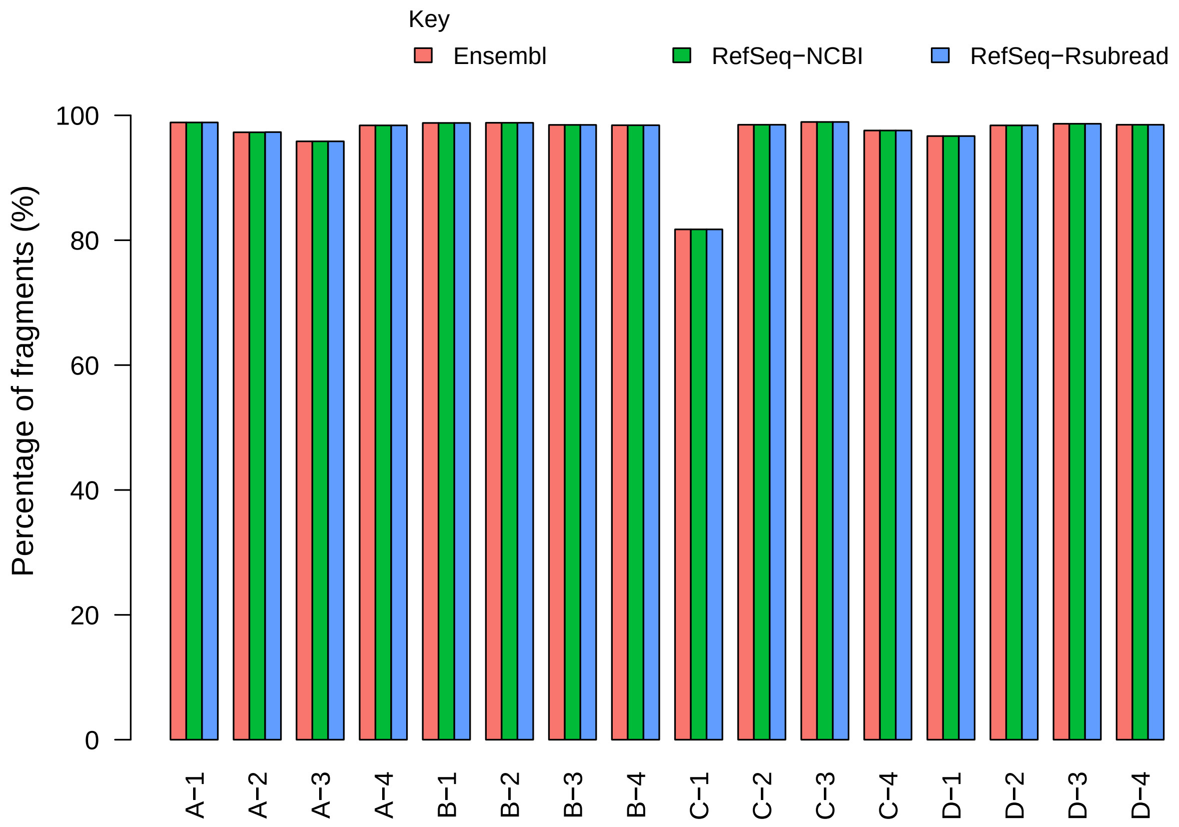

Figure S4. Percentage of fragments that were successfully aligned to the human reference genome GRCh38 in each library.

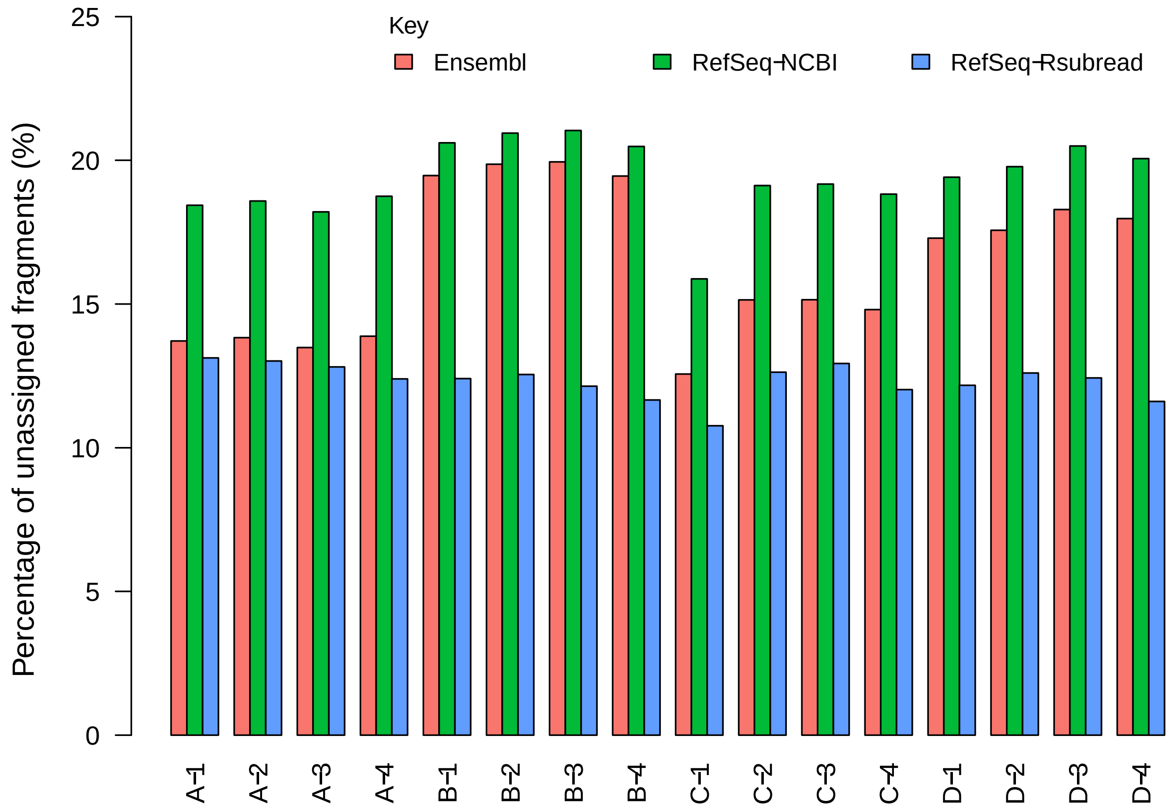

Figure S5. Percentages of fragments that failed to be assigned because they did not hit any annotated exons in an annotation. The percentage is calculated as the number of unassigned fragments divided by all the fragments included in a library.

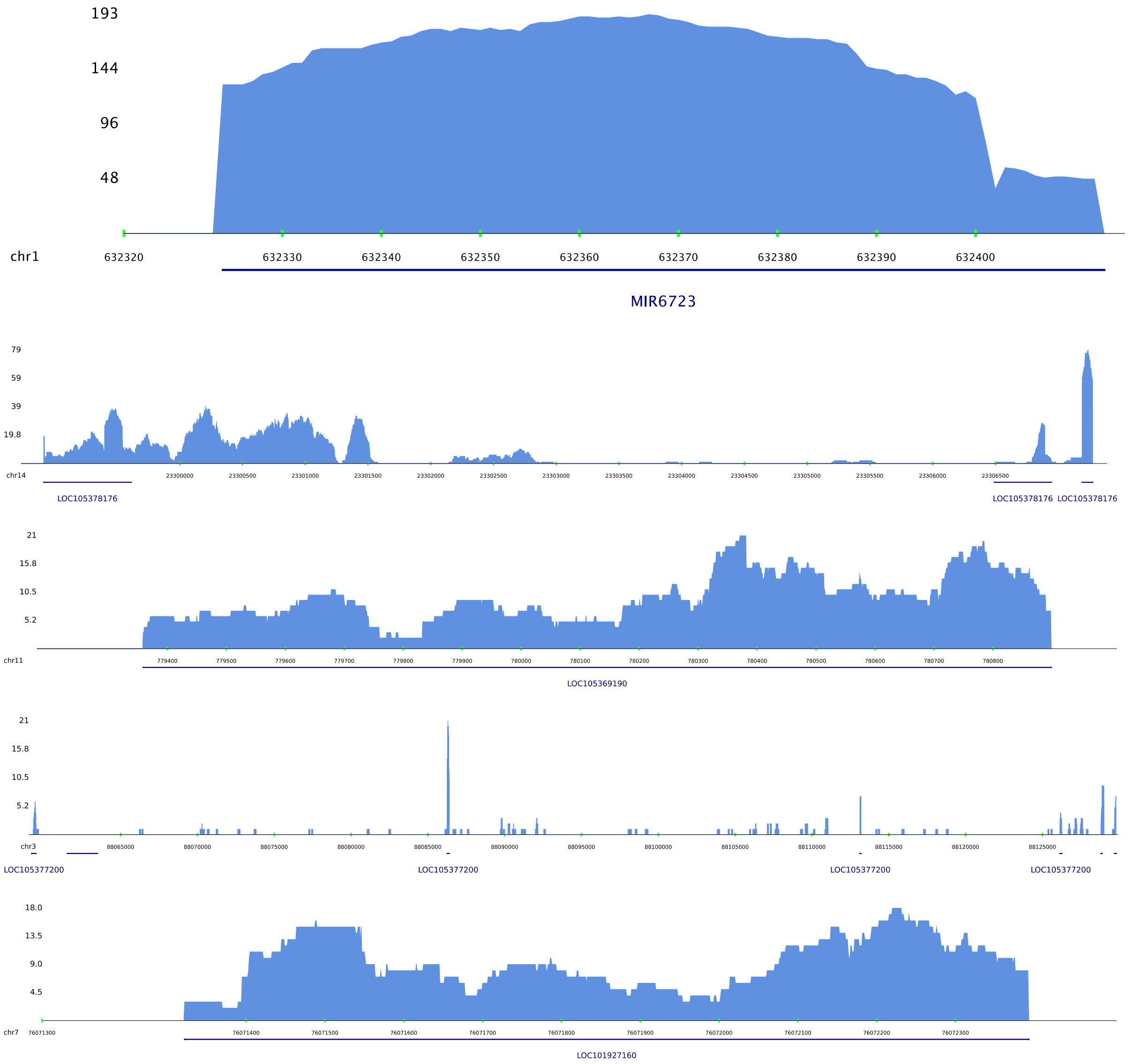

Figure S6. Read mapping results for selected genes that are present in the RefSeq-Rsubread annotation but not in the RefSeq-NCBI and Ensembl annotations. The top three tracks contain data from a Human Brain Reference RNA sample (library B1), and the bottom two tracks contain data from a Universal Human Reference RNA sample (library A1). The vertical axis shows the number of mapped fragments. Bars under the chromosomal coordinates at the horizontal axis represent annotated exons of a gene.

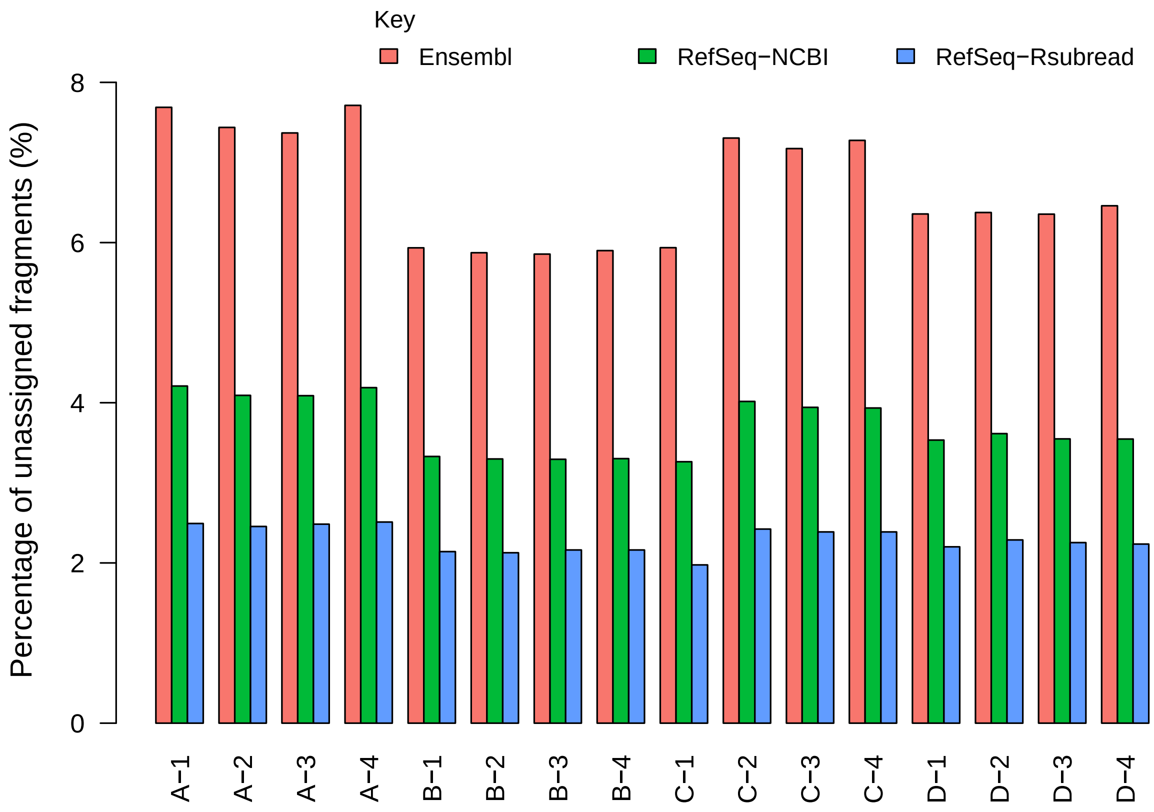

Figure S7. Percentages of fragments that failed to be assigned because of overlapping more than one gene in an annotation. The percentage is calculated as the number of unassigned fragments divided by all the fragments included in a library.

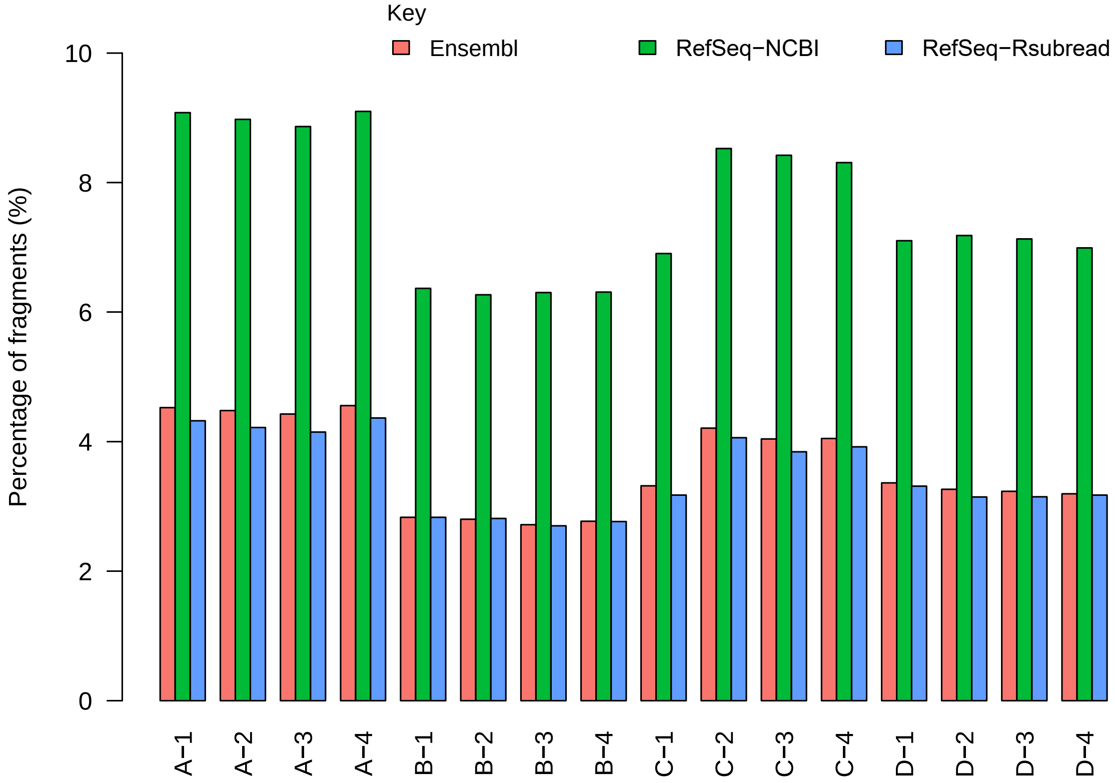

Figure S8. Percentage of multi-mapping fragments in each library. Multi-mapping fragments are those fragments that can equally best map to more than one location in the genome. However, the percentage of such fragments in a library is affected by the choice of gene annotation provided to the Subread aligner for mapping. The Subread aligner tries to use the gene annotation data to break tie when multiple best mapping locations were discovered for a fragment.

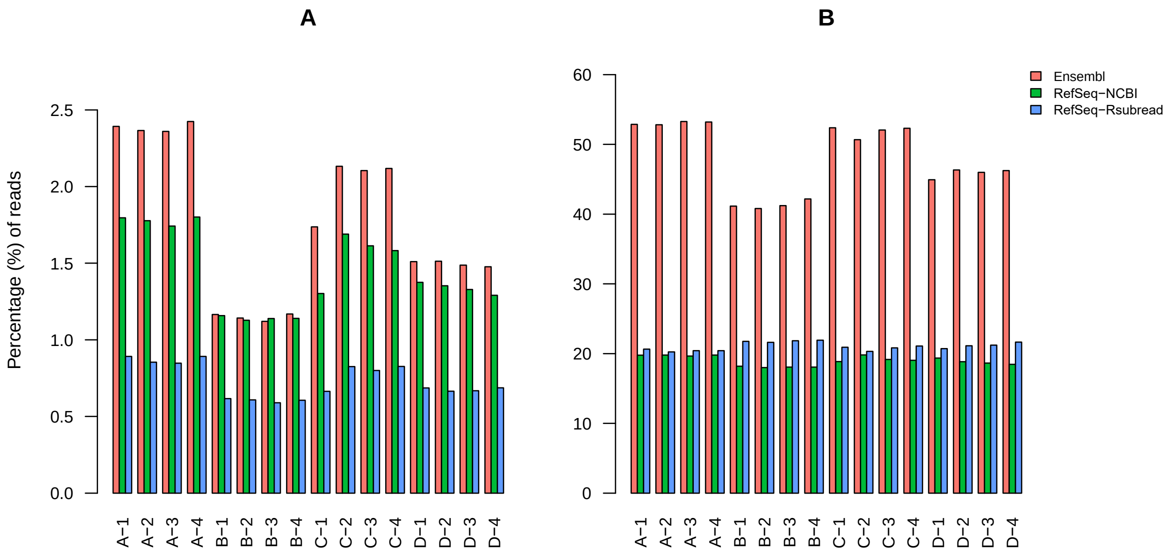

Figure S9. Barplots showing percentages of multi-mapping fragments that overlapped more than one gene. (A) Percentage of such fragments in all the fragments included in each library. (B) Percentage of such fragments in all the multi-mapping fragments found in each library.

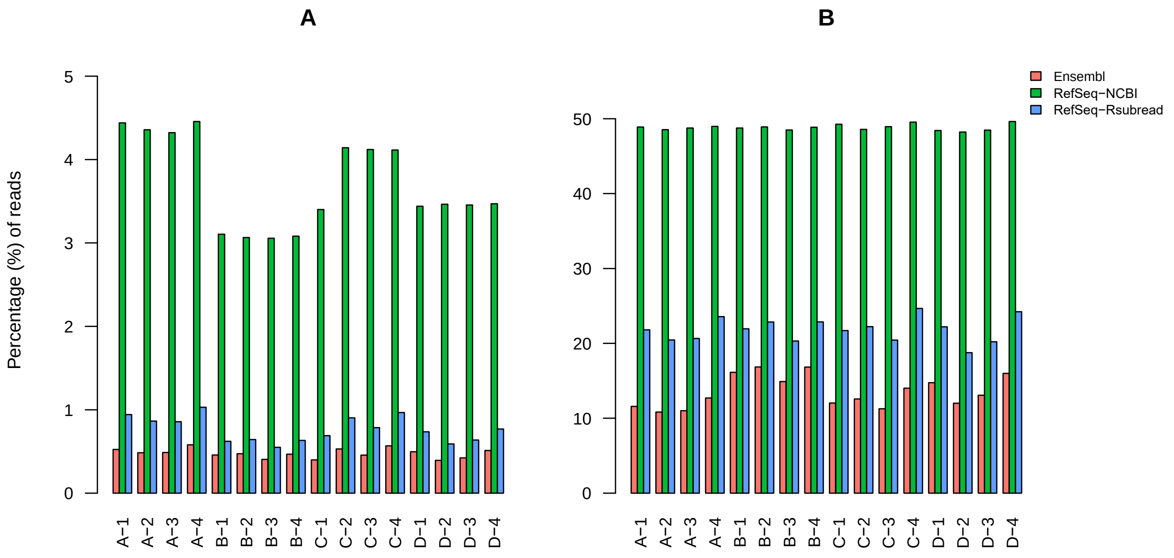

Figure S10. Barplots showing percentages of multi-mapping fragments that overlapped one or more exons within the same gene. (A) Percentage of such fragments in all the fragments included in each library. (B) Percentage of such fragments in all the multi-mapping fragments found in each library.

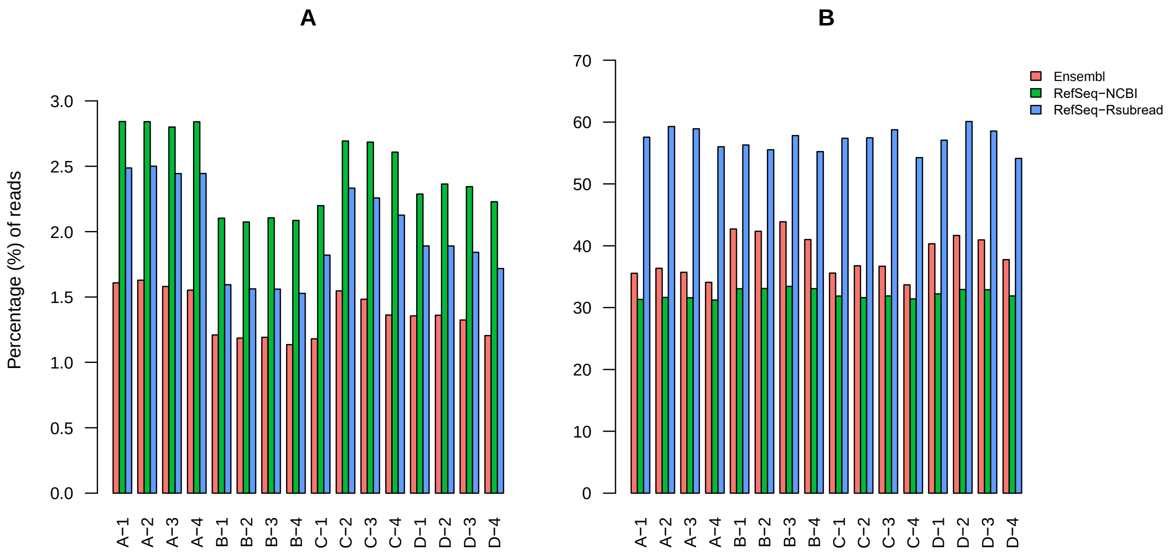

Figure S11. Barplots showing percentages of multi-mapping fragments that did not overlap any exon in any gene. (A) Percentage of such fragments in all the fragments included in each library. (B) Percentage of such fragments in all the multi-mapping fragments found in each library.

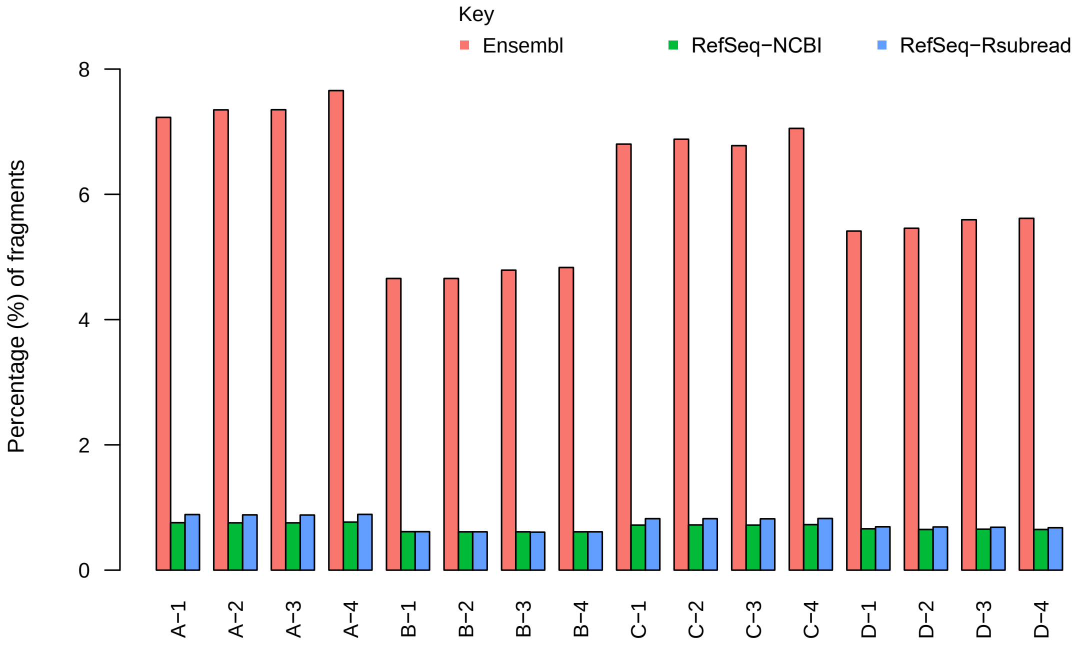

Figure S12. Percentage of fragments assigned to pseudogenes in each library with different annotations used for mapping and counting. Note that the Ensembl annotation contains a lot more pseudogenes that the RefSeq-NCBI and RefSeq-Rsubread annotations.

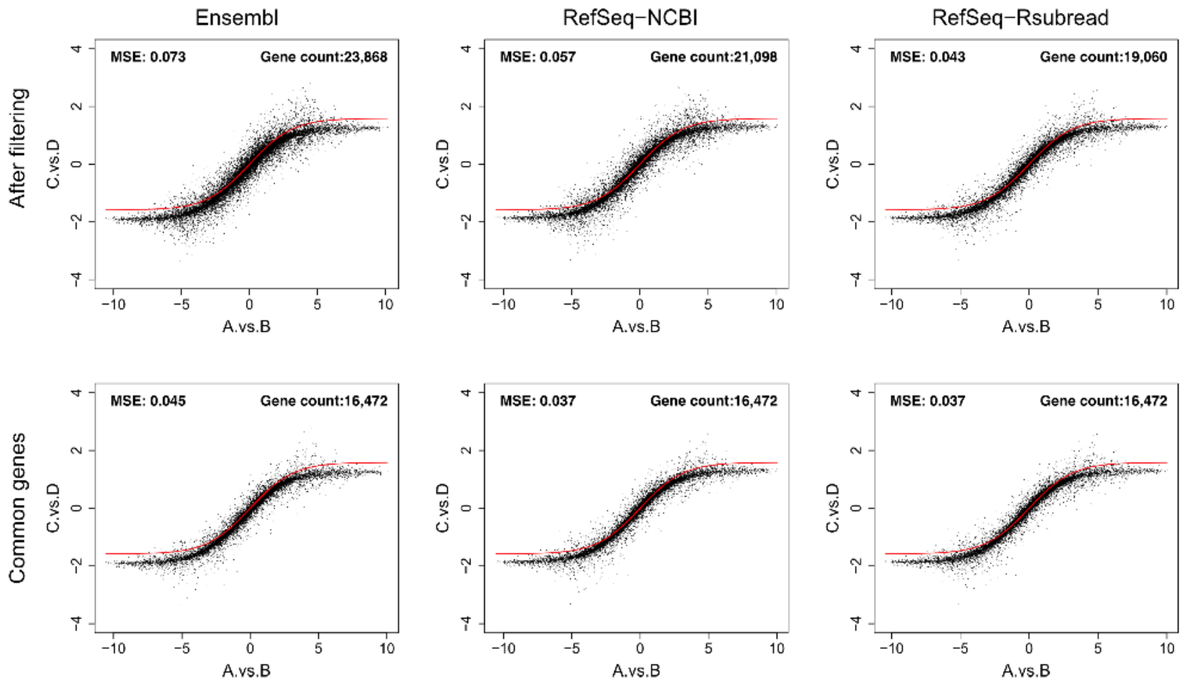

Figure S13. Titration monotonicity plots generated from using TMM normalized data. The red curve in each plot represents the reference titration. The Mean Squared Error (MSE) between the reference titration and the actual titration was calculated for each annotation, using all the genes that remained after filtering for lowly expressed genes (top row) or using common genes between the three annotations after filtering for lowly expressed genes (bottom row). In each plot, the horizontal axis represents the $\log_2$ fold changes of gene expression between sample A and sample B and the vertical axis represents the $\log_2$ fold changes of gene expression between sample C and sample D.

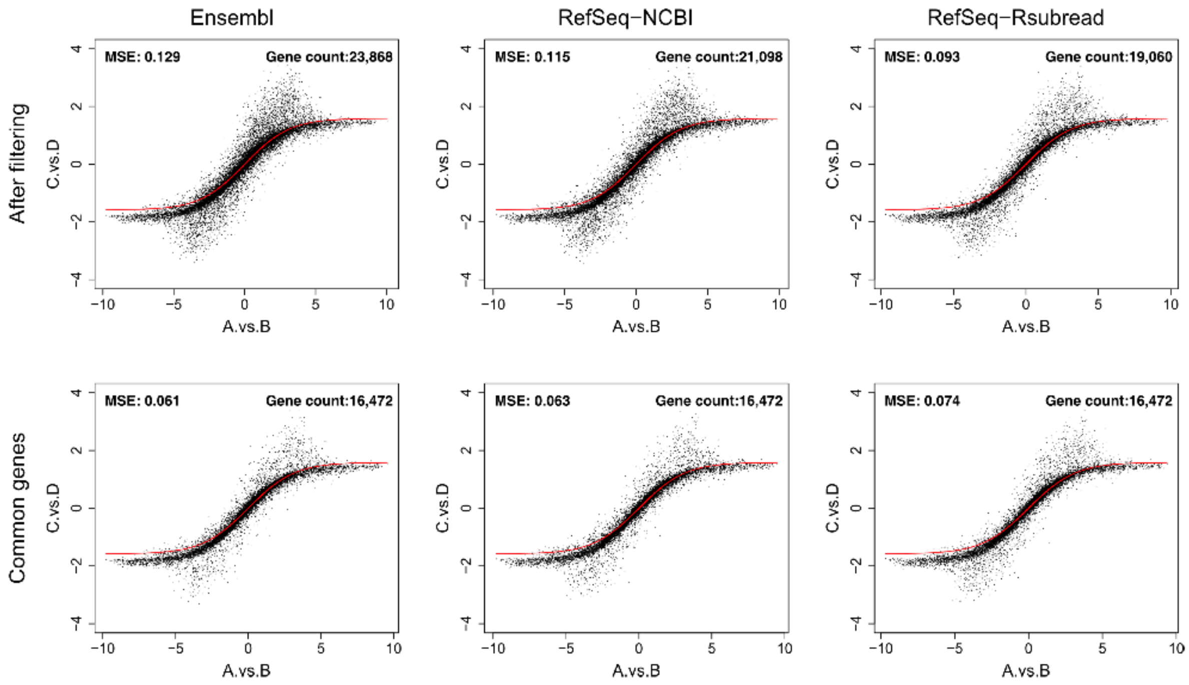

Figure S14. Titration monotonicity plots generated from using quantile normalized data. The red curve in each plot represents the reference titration. The Mean Squared Error (MSE) between the reference titration and the actual titration was calculated for each annotation, using all the genes that remained after filtering for lowly expressed genes (top row) or using common genes between the three annotations after filtering for lowly expressed genes (bottom row). In each plot, the horizontal axis represents the $\log_2$ fold changes of gene expression between sample A and sample B and the vertical axis represents the $\log_2$ fold changes of gene expression between sample C and sample D.

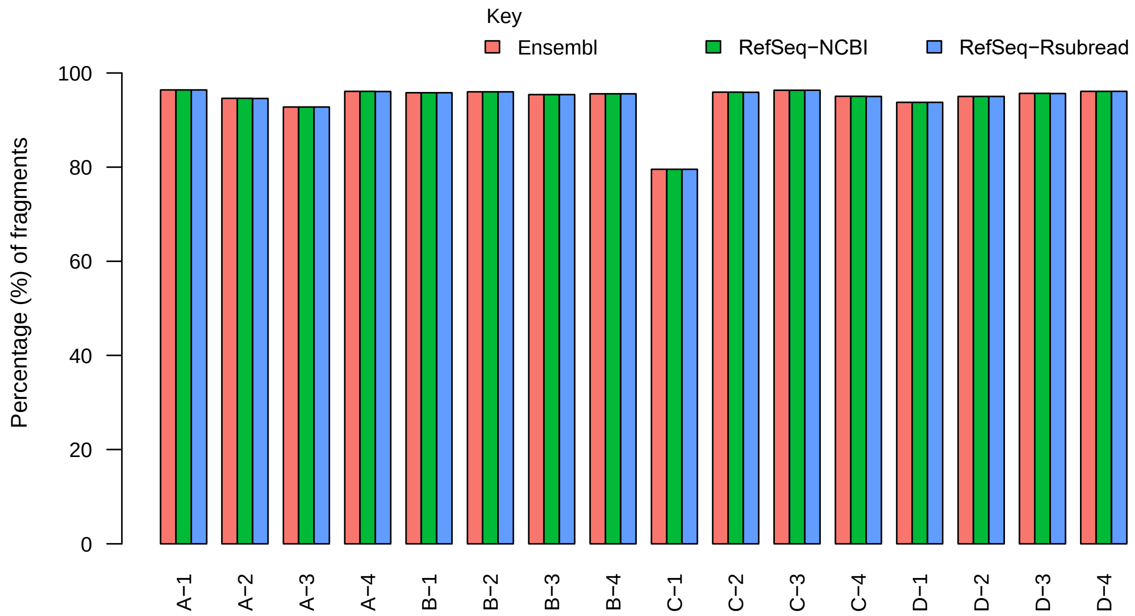

Figure S15. Percentage of fragments that were successfully aligned to the human reference genome GRCh38 by the STAR aligner.

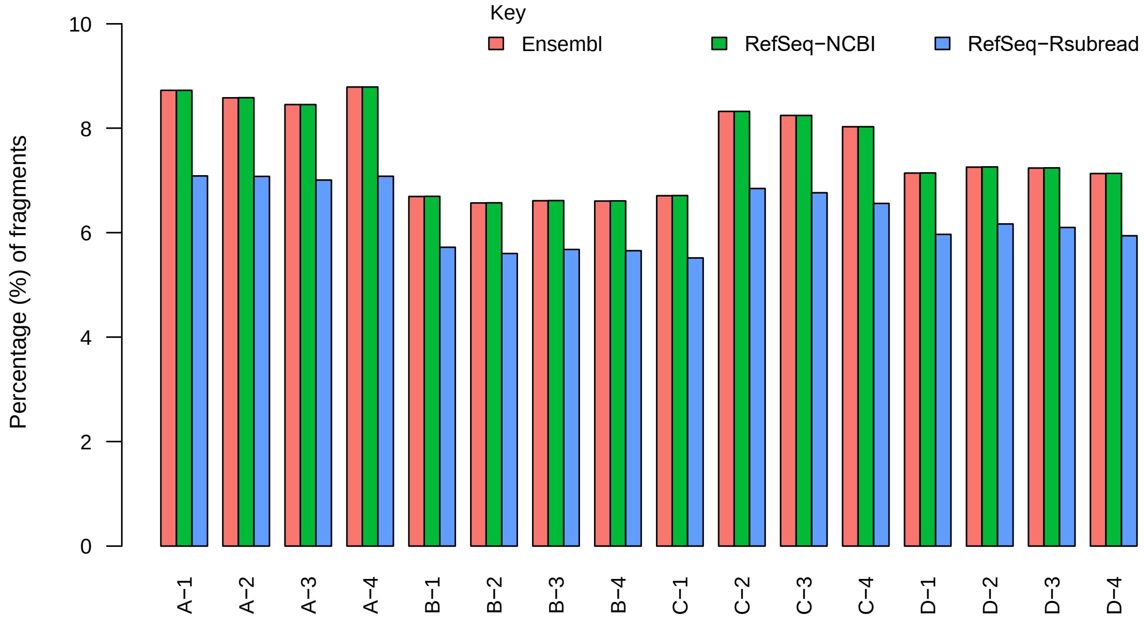

Figure S16. Percentage of multi-mapping fragments that were reported by STAR aligner.

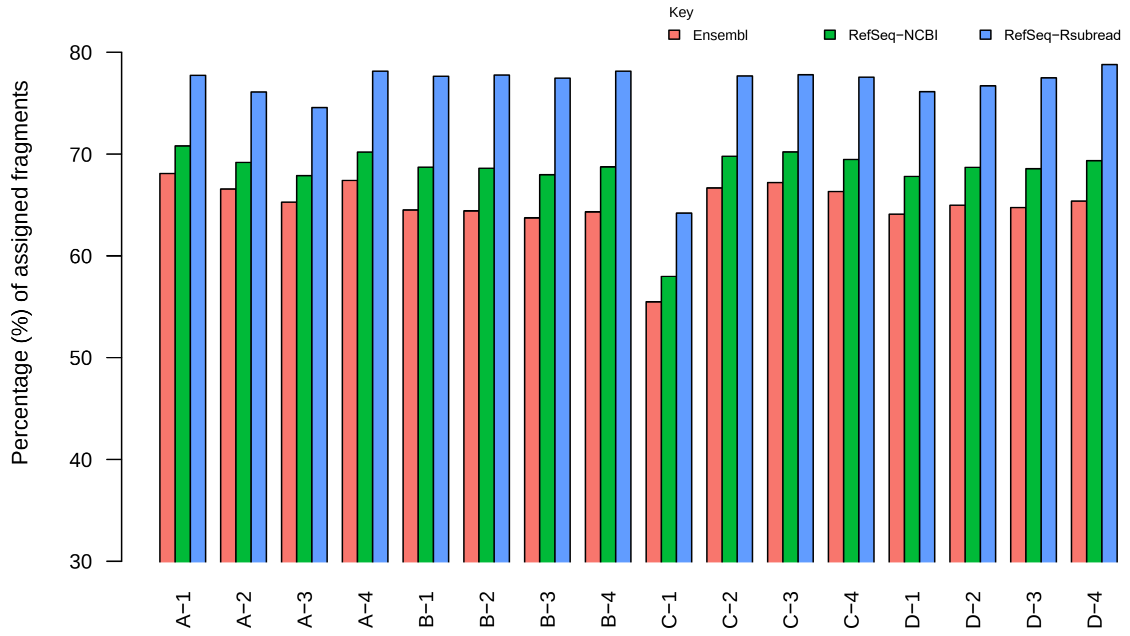

Figure S17. Percentages of fragments successfully assigned to genes in each annotation from using the STAR mapping results.

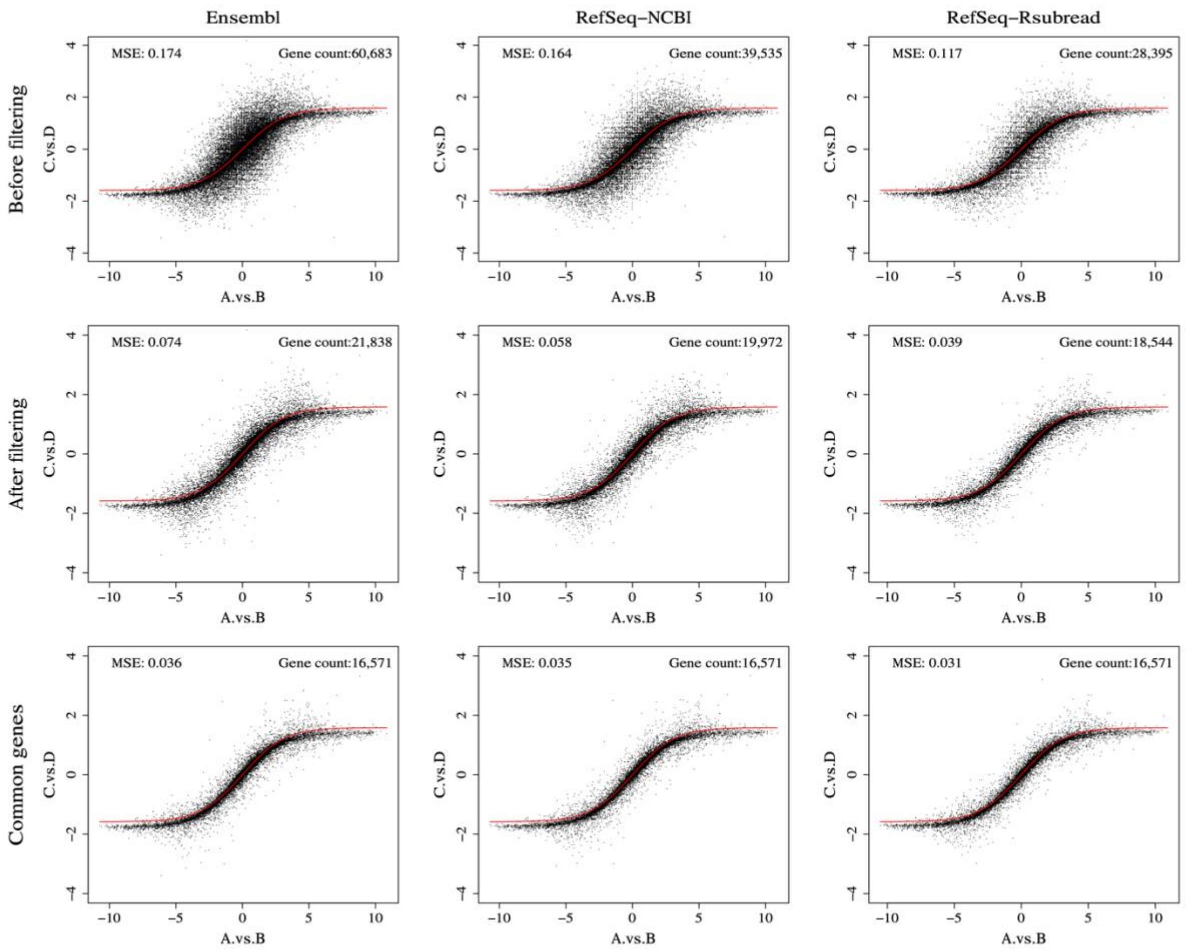

Figure S18. Titration monotonicity plots generated from using the STAR mapping results. Data were library size normalized.

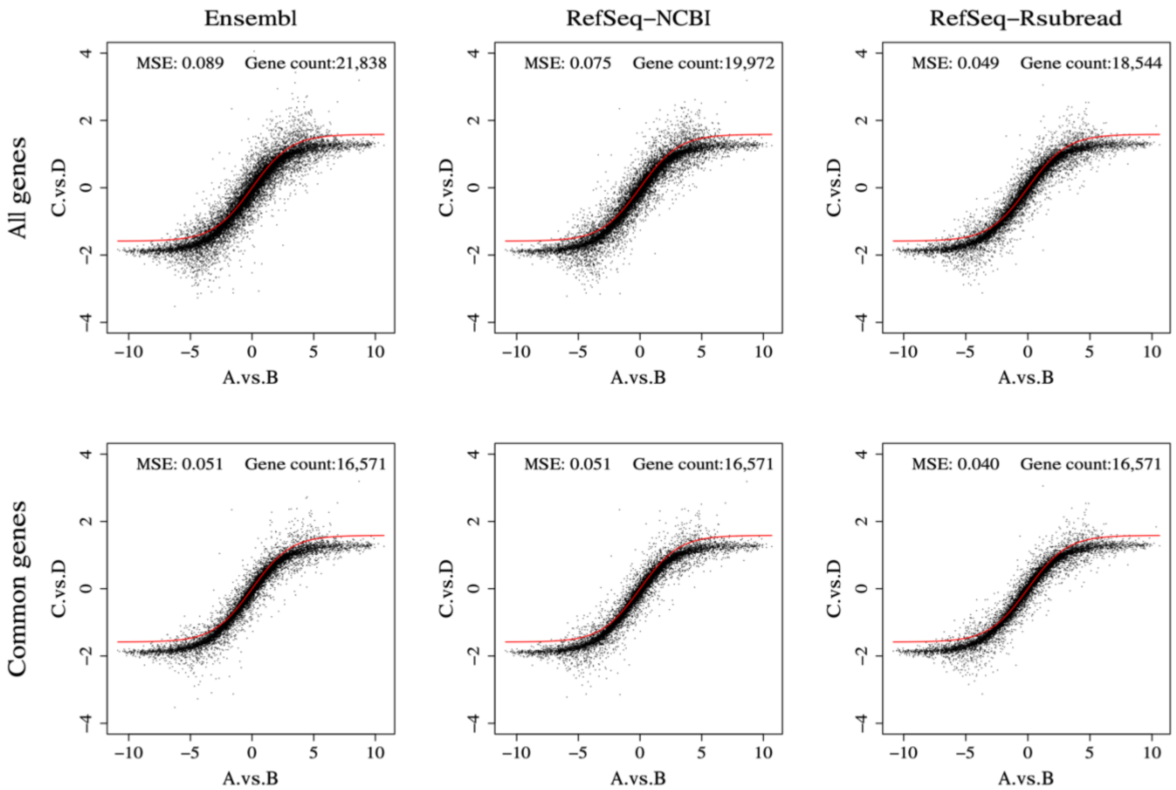

Figure S19. Titration monotonicity plots generated from using the STAR mapping results. Data were TMM normalized.

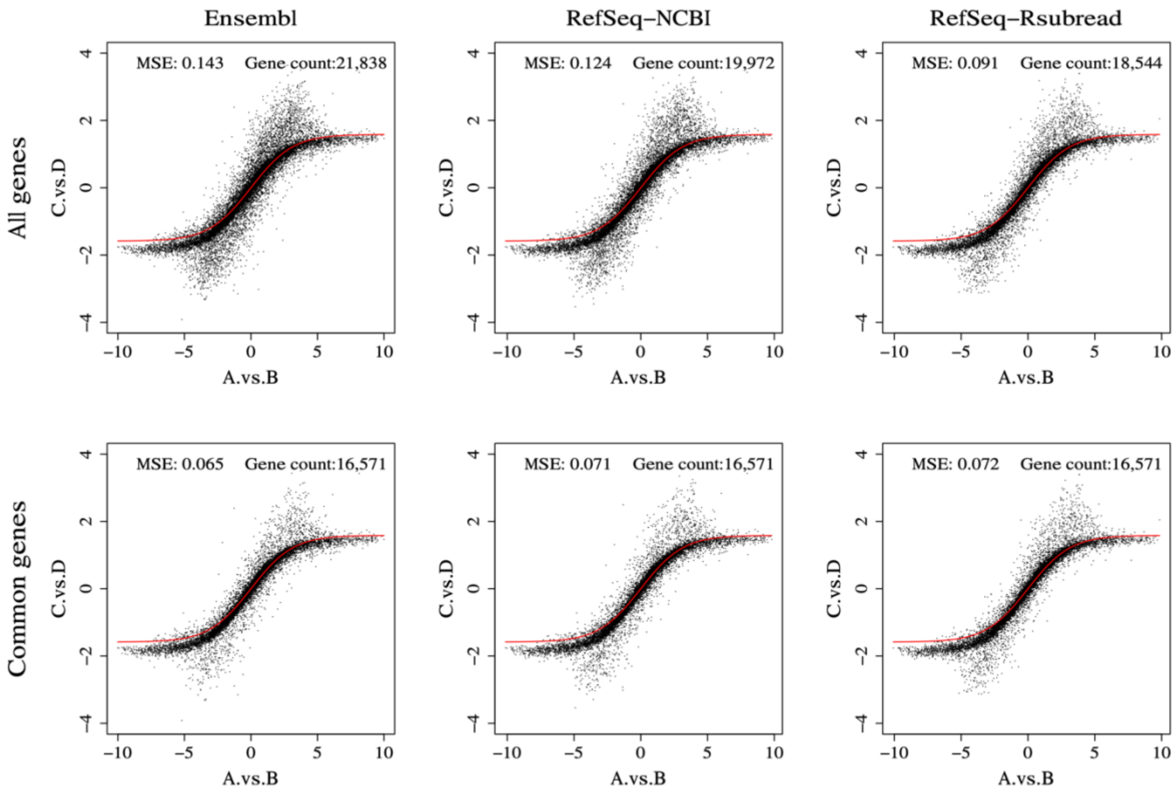

Figure S20. Titration monotonicity plots generated from using the STAR mapping results. Data were quantile normalized.

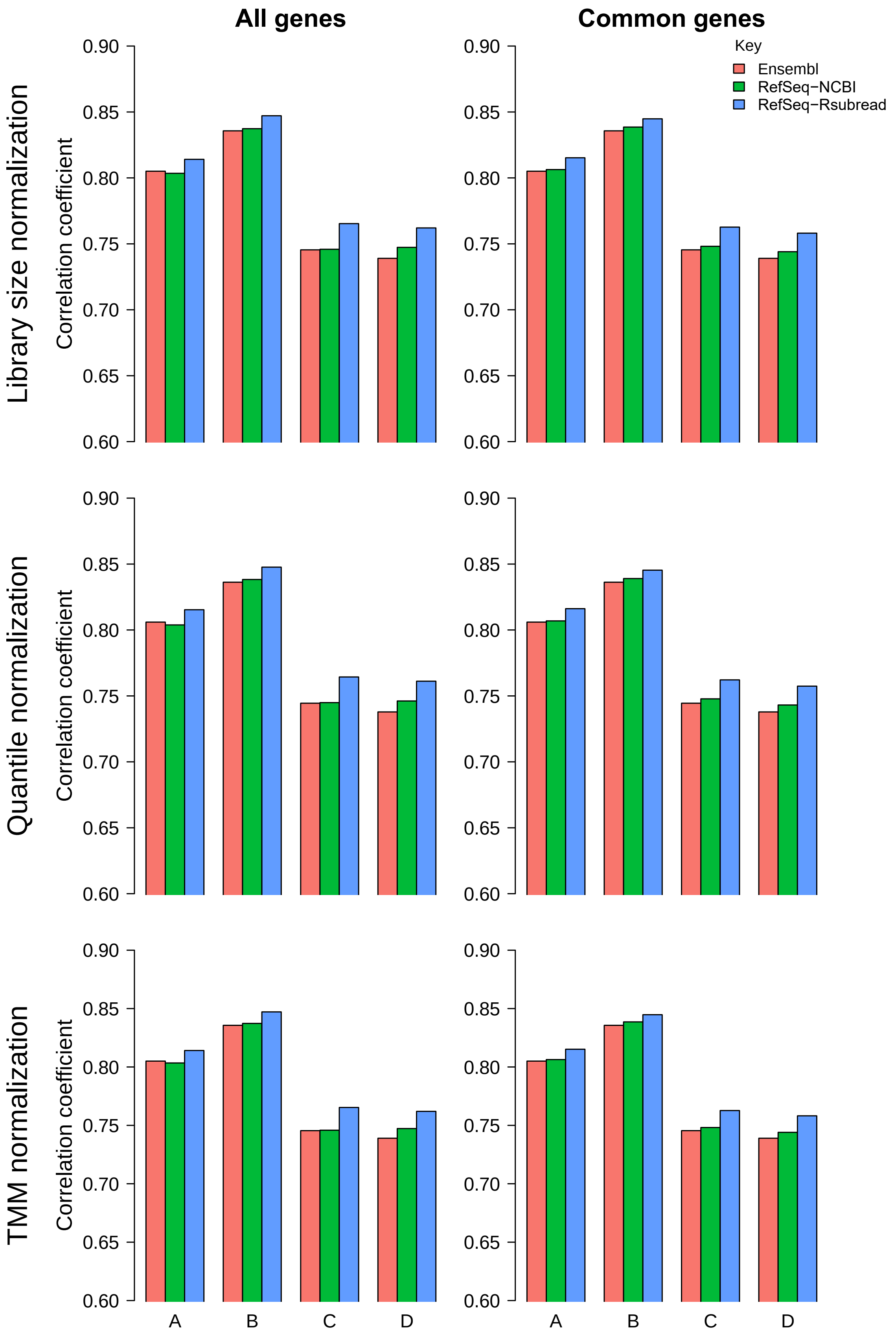

Figure S21. Pearson correlation analysis to compare RNA-seq data against TaqMan RT-PCR data. The analysis was performed based on the STAR mapping results.

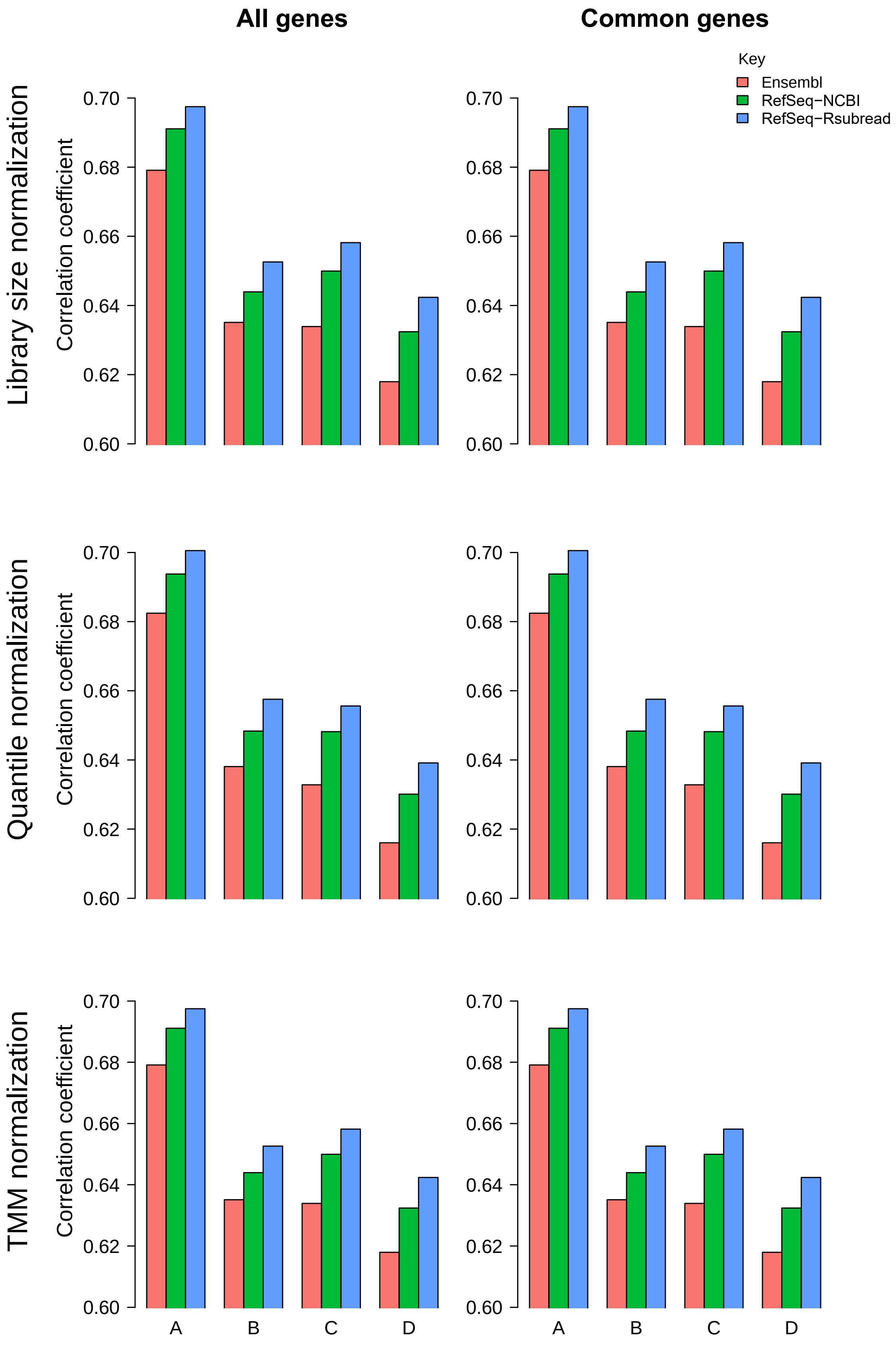

Figure S22. Pearson correlation analysis to compare RNA-seq data against microarray data. The analysis was performed based on the STAR mapping results.

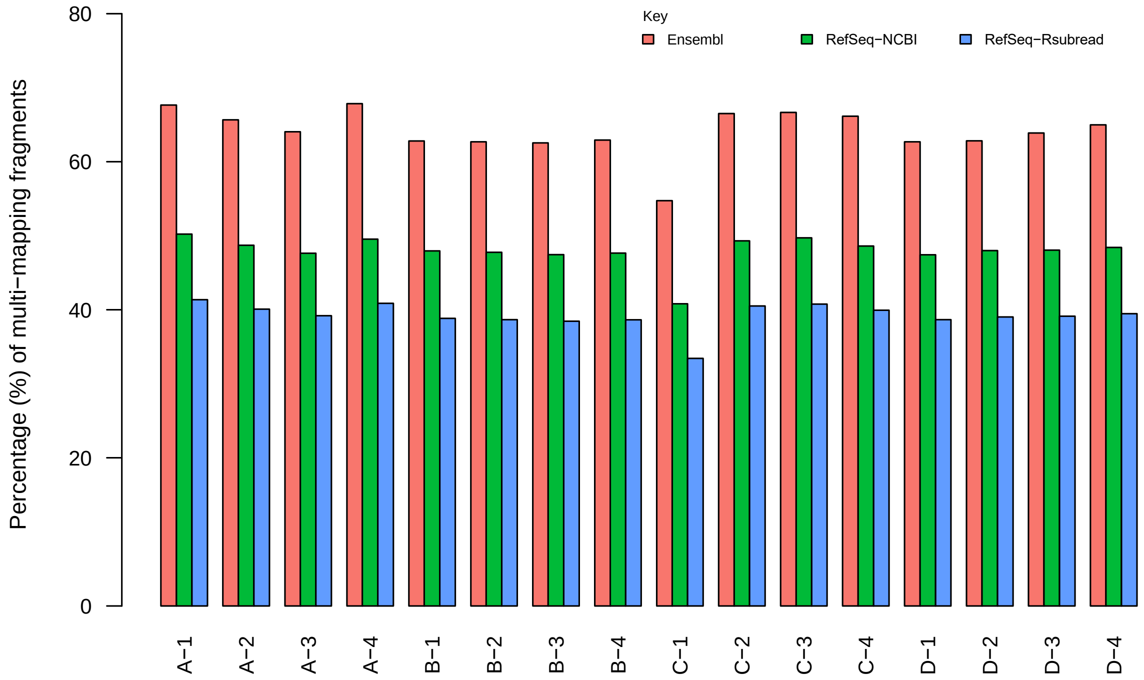

Figure S23. Barplots showing percentages of fragments that mapped to more than one transcript in each library in the SEQC dataset by RSEM.

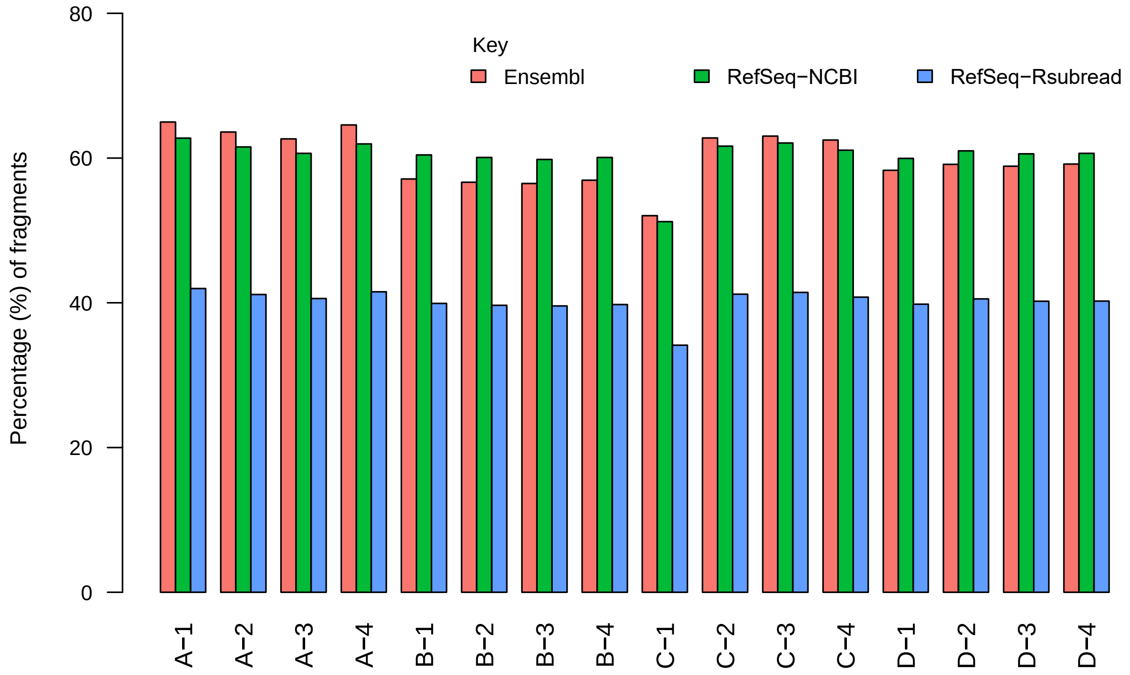

Figure S24. Barplots showing percentages of fragments that were pseudo-aligned to more than one transcript in each library in the SEQC dataset by Kallisto.

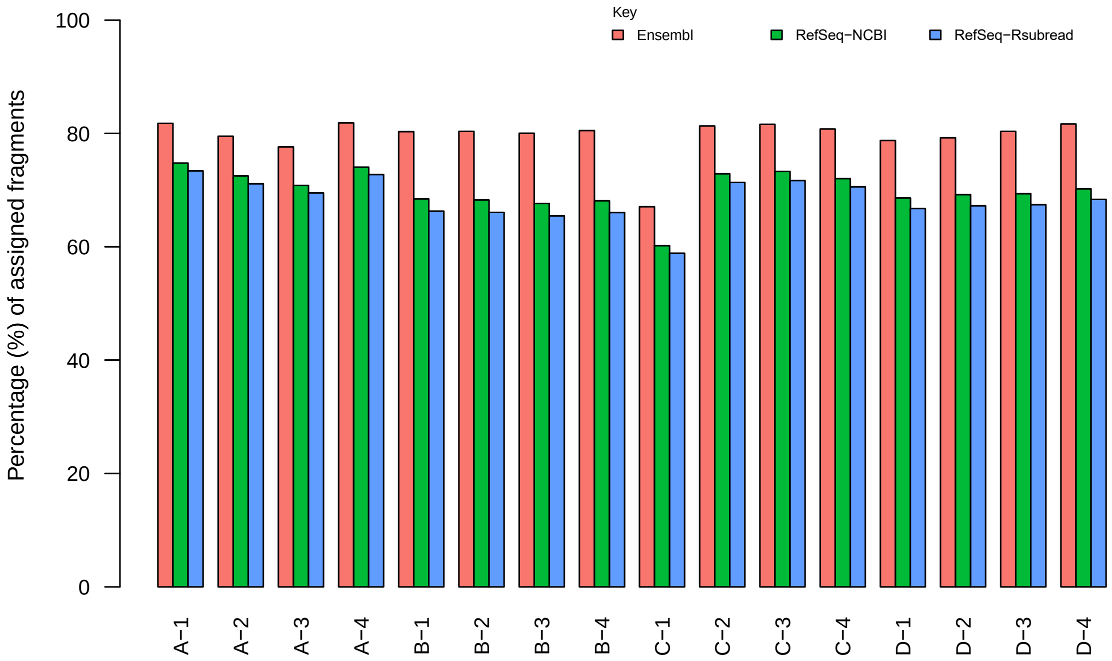

Figure S25. Barplots showing percentages of fragments that were assigned to transcripts in each library in the SEQC dataset by RSEM.

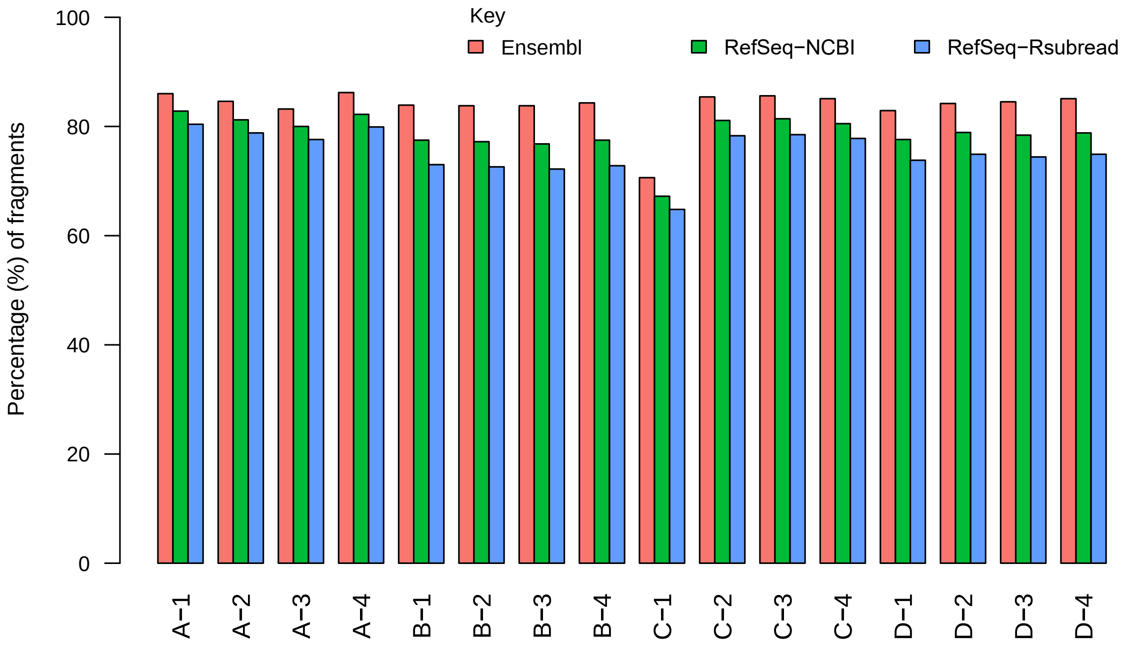

Figure S26. Barplots showing percentages of fragments that were assigned to transcripts in each library in the SEQC dataset by Kallisto.

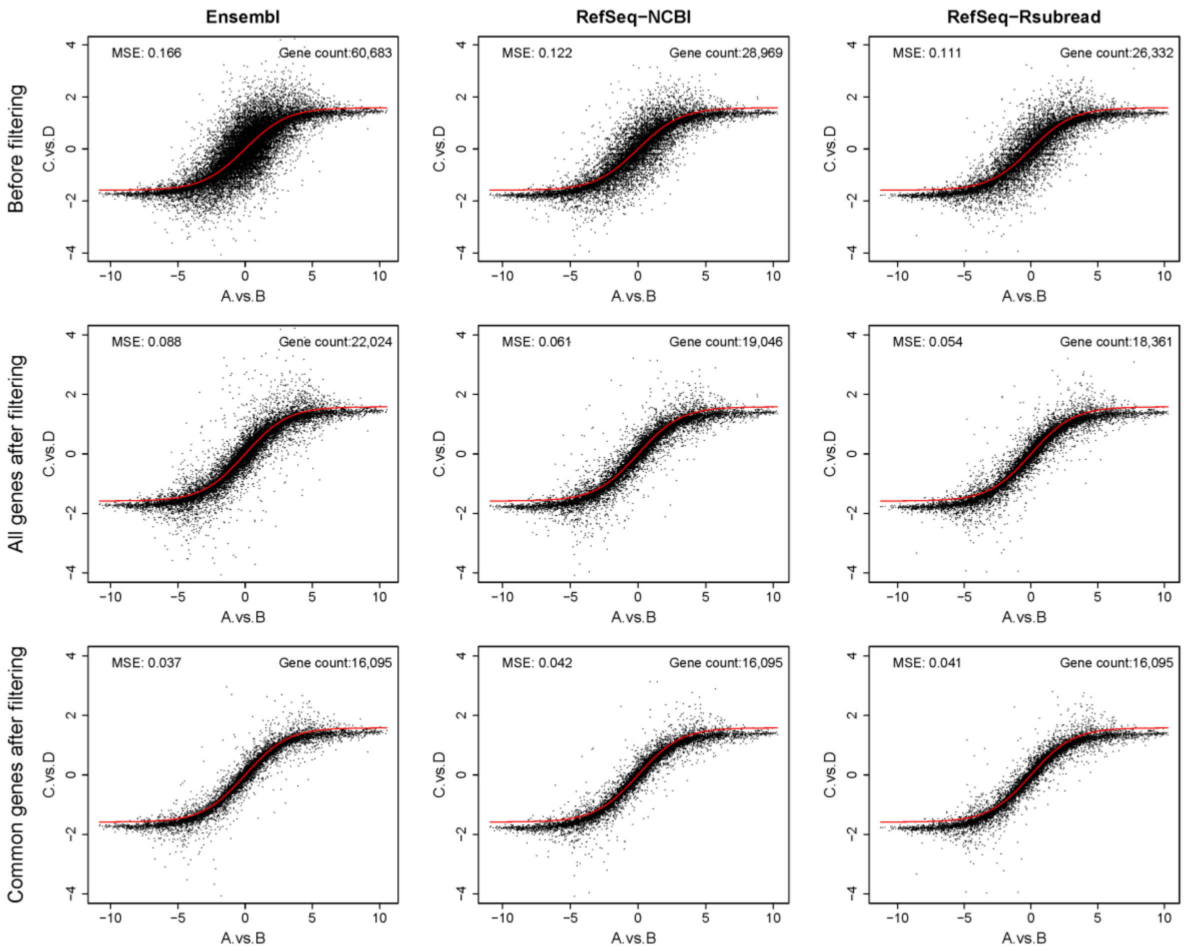

Figure S27. Titration monotonicity plots generated from using the RSEM quantification results. Data were library size normalized.

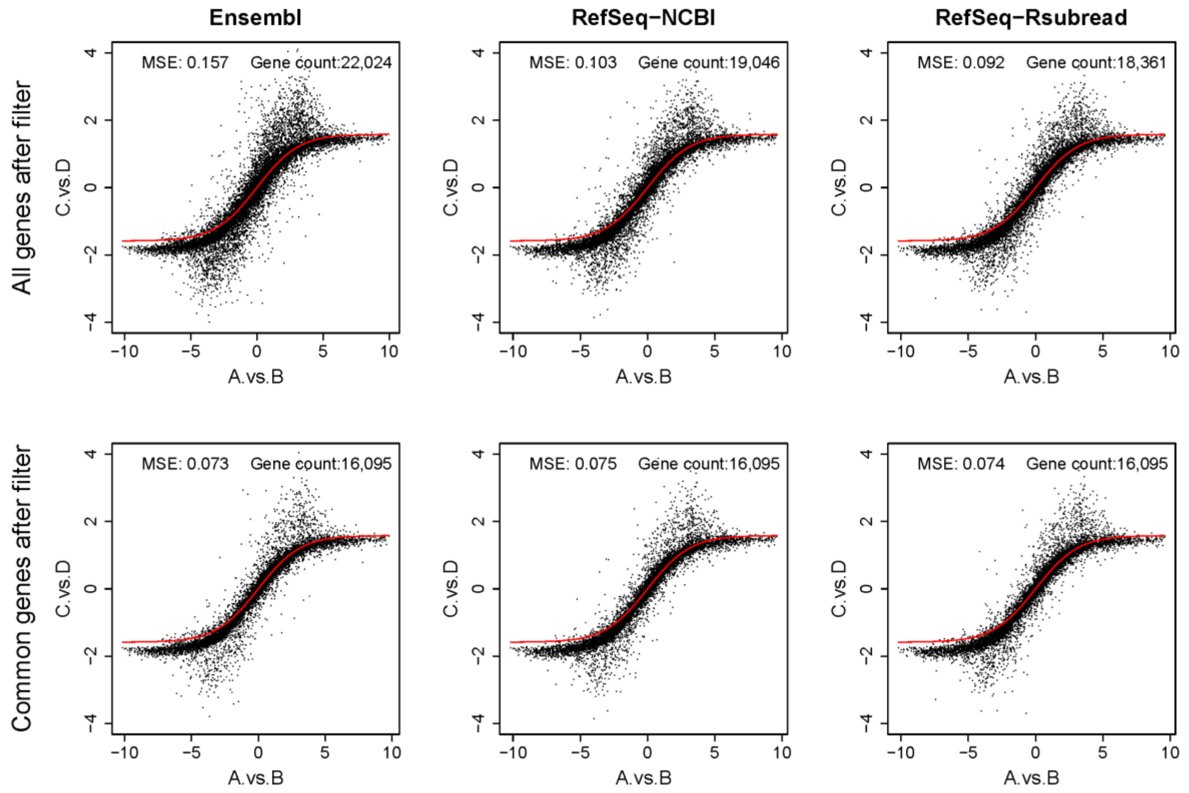

Figure S28. Titration monotonicity plots generated from using the RSEM quantification results. Data were quantile normalized.

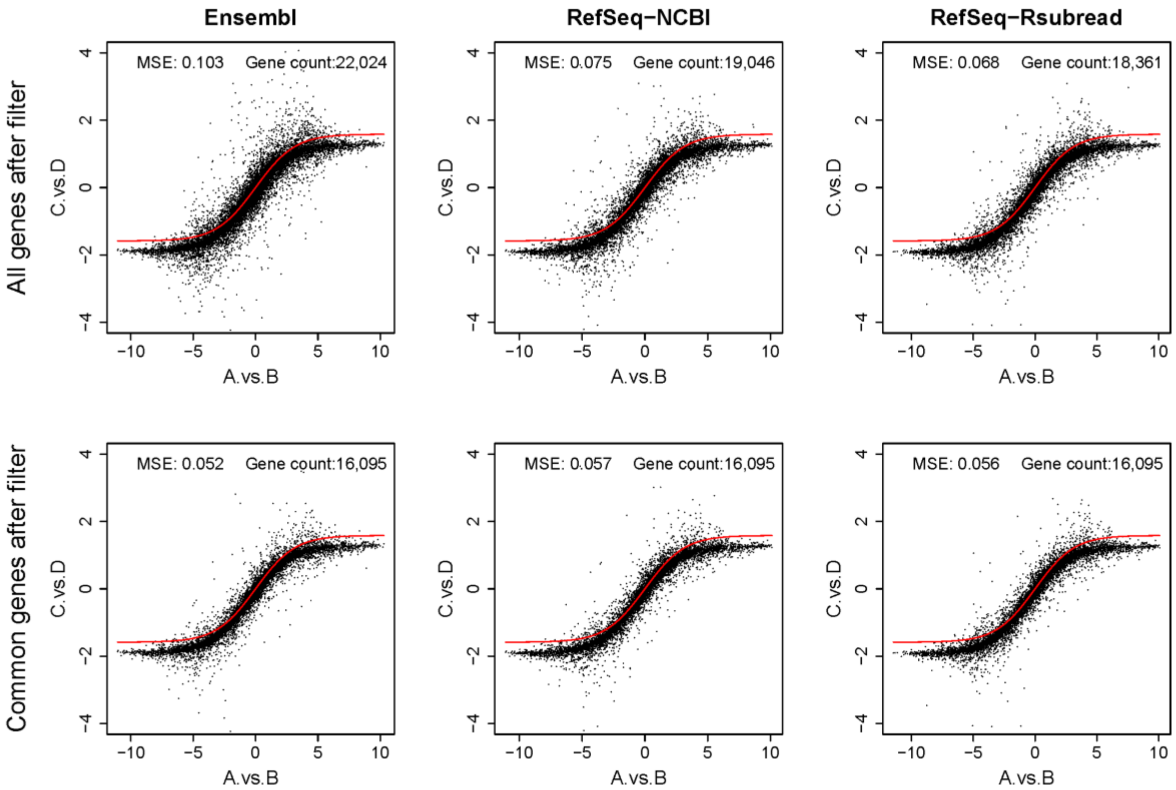

Figure S29. Titration monotonicity plots generated from using the RSEM quantification results. Data were TMM normalized.

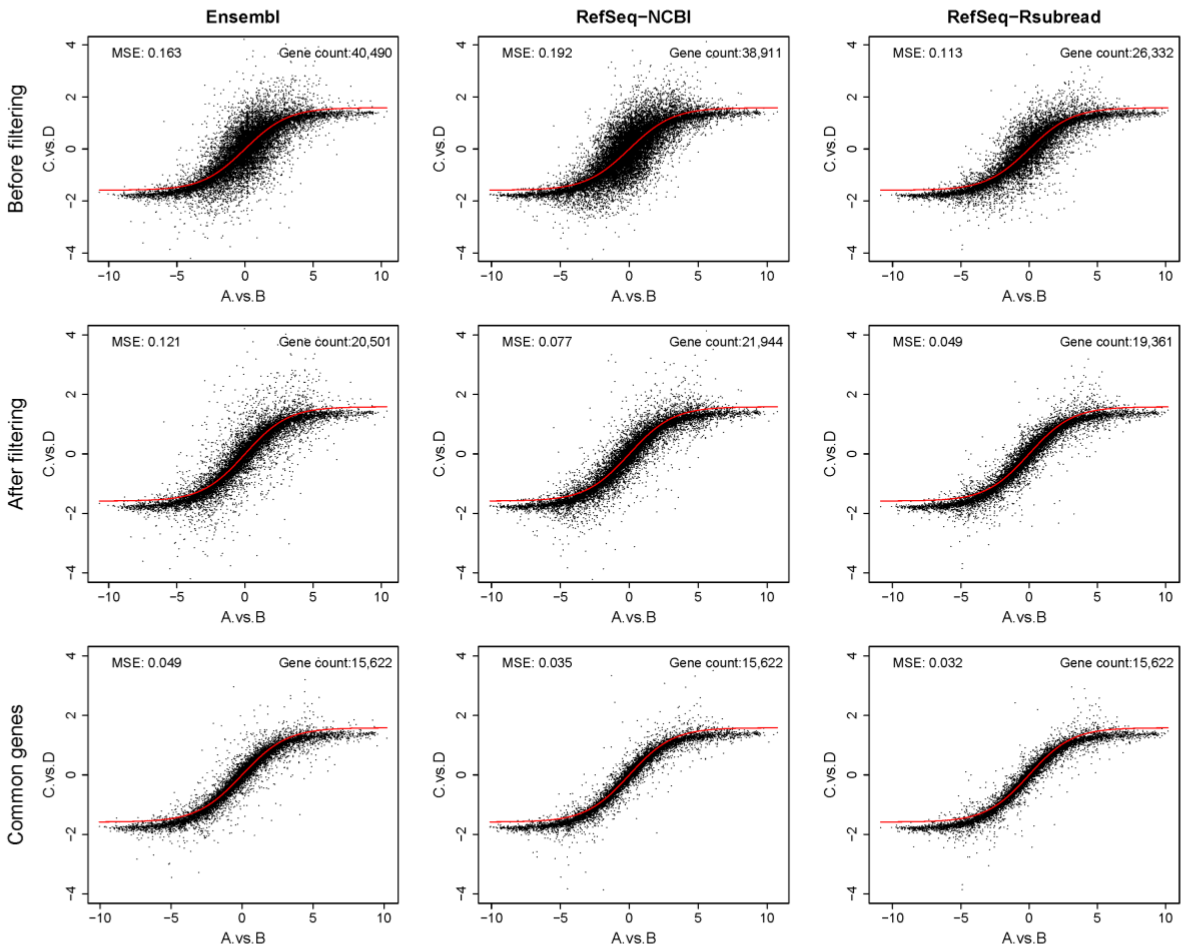

Figure S30. Titration monotonicity plots generated from using the Kallisto quantification results. Data were library size normalized.

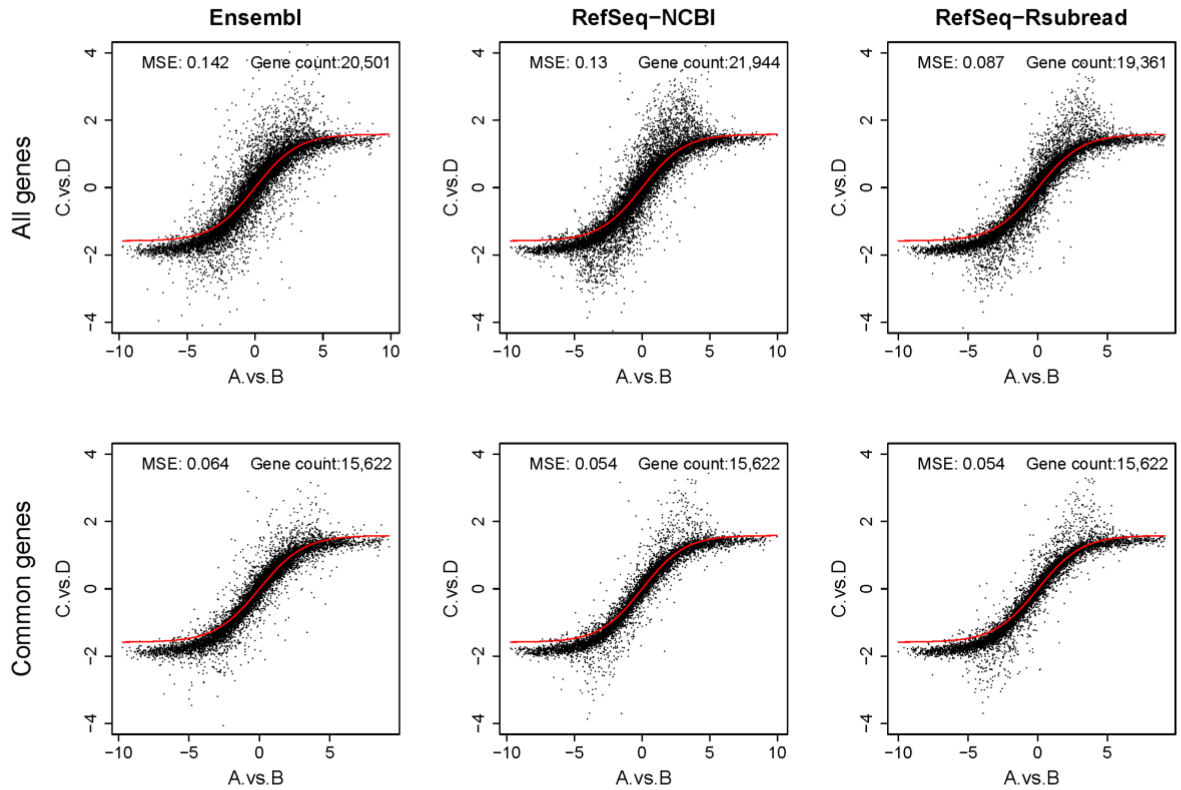

Figure S31. Titration monotonicity plots generated from using the Kallisto quantification results. Data were quantile normalized.

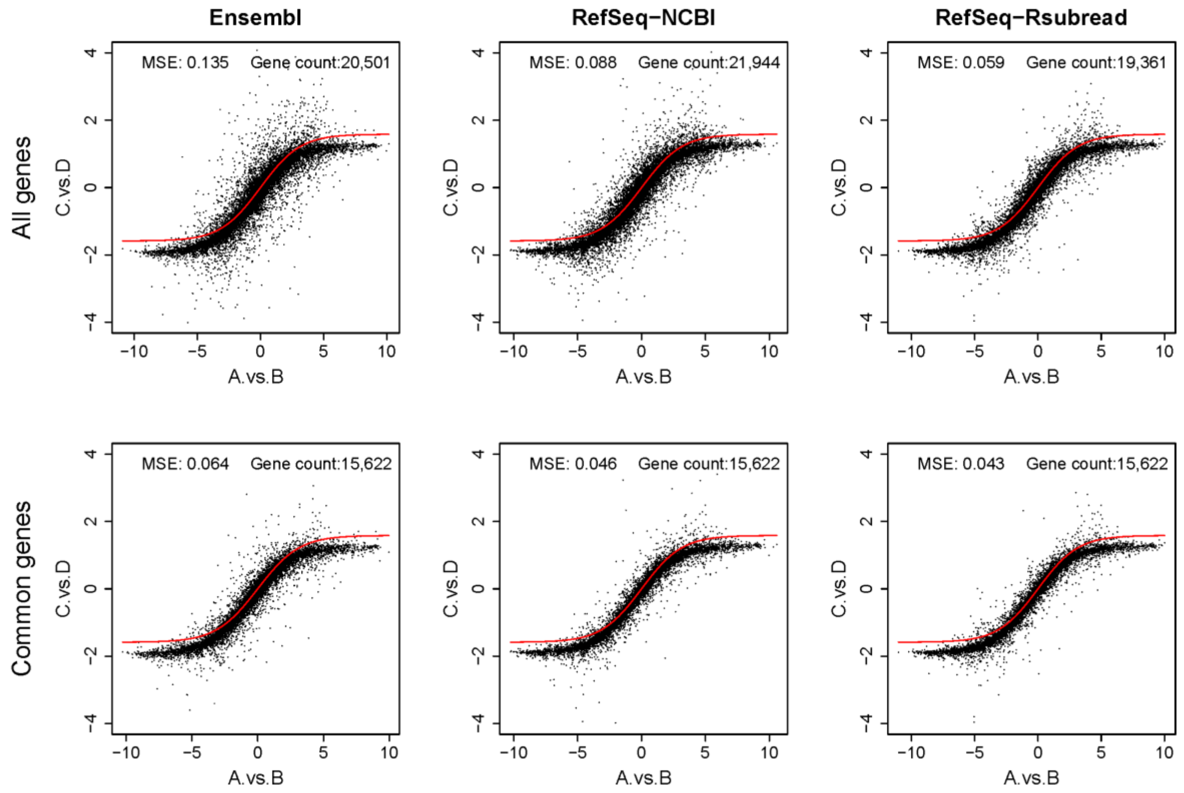

Figure S32. Titration monotonicity plots generated from using the Kallisto quantification results. Data were TMM normalized.

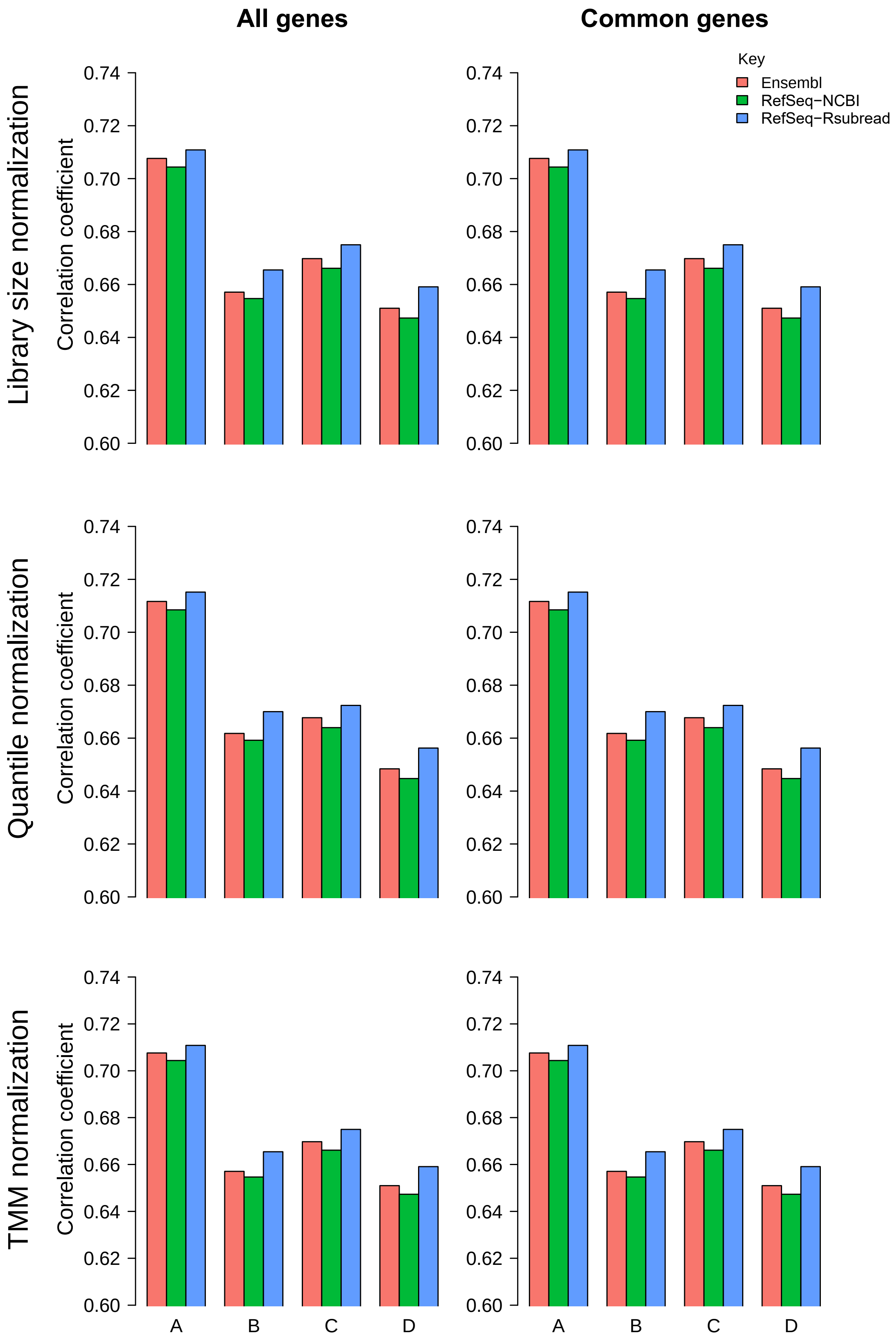

Figure S33. Pearson correlation analysis to compare RNA-seq data against microarray data. The analysis was performed based on the RSEM quantification results.

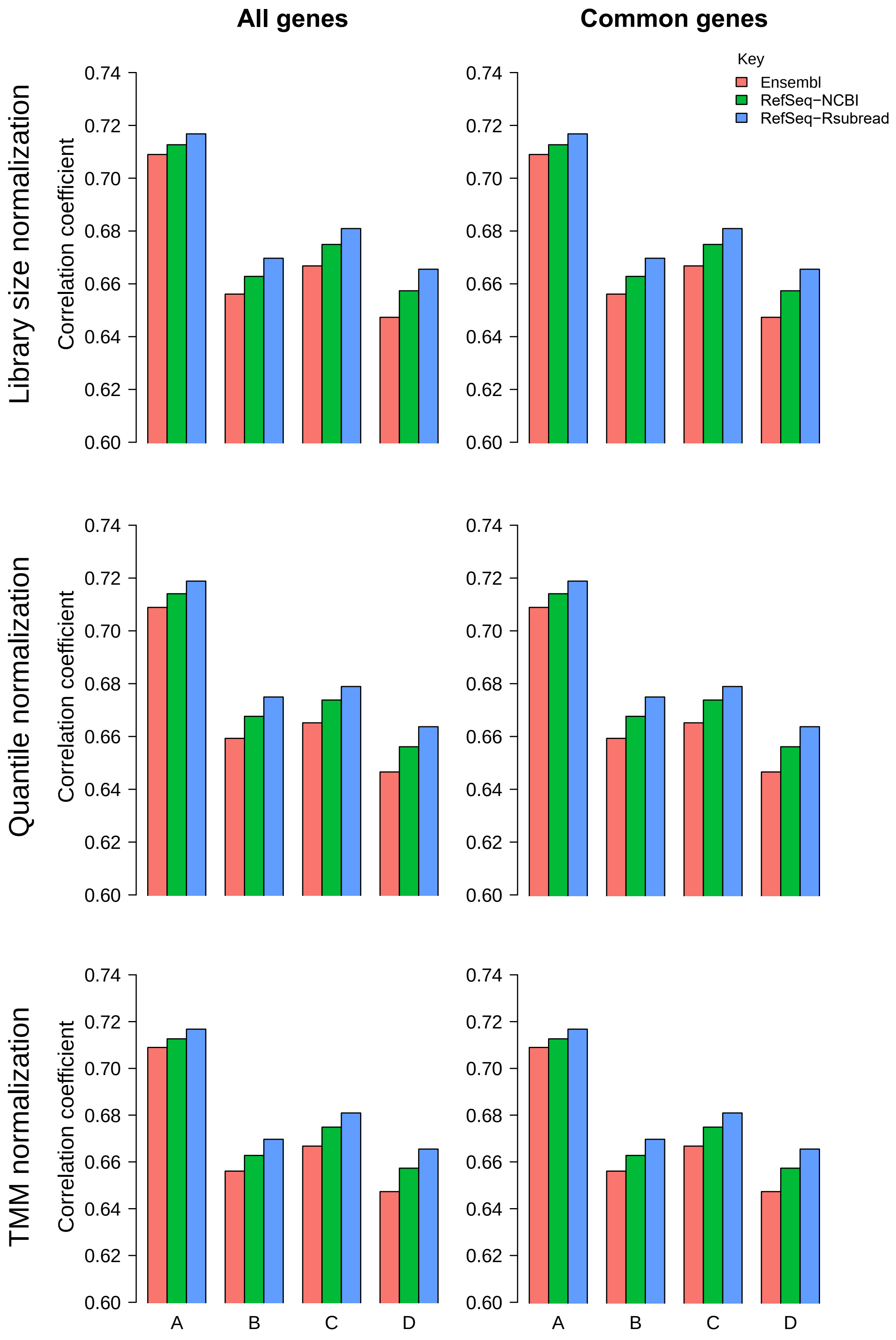

Figure S34. Pearson correlation analysis to compare RNA-seq data against microarray data. The analysis was performed based on the Kallisto quantification results.

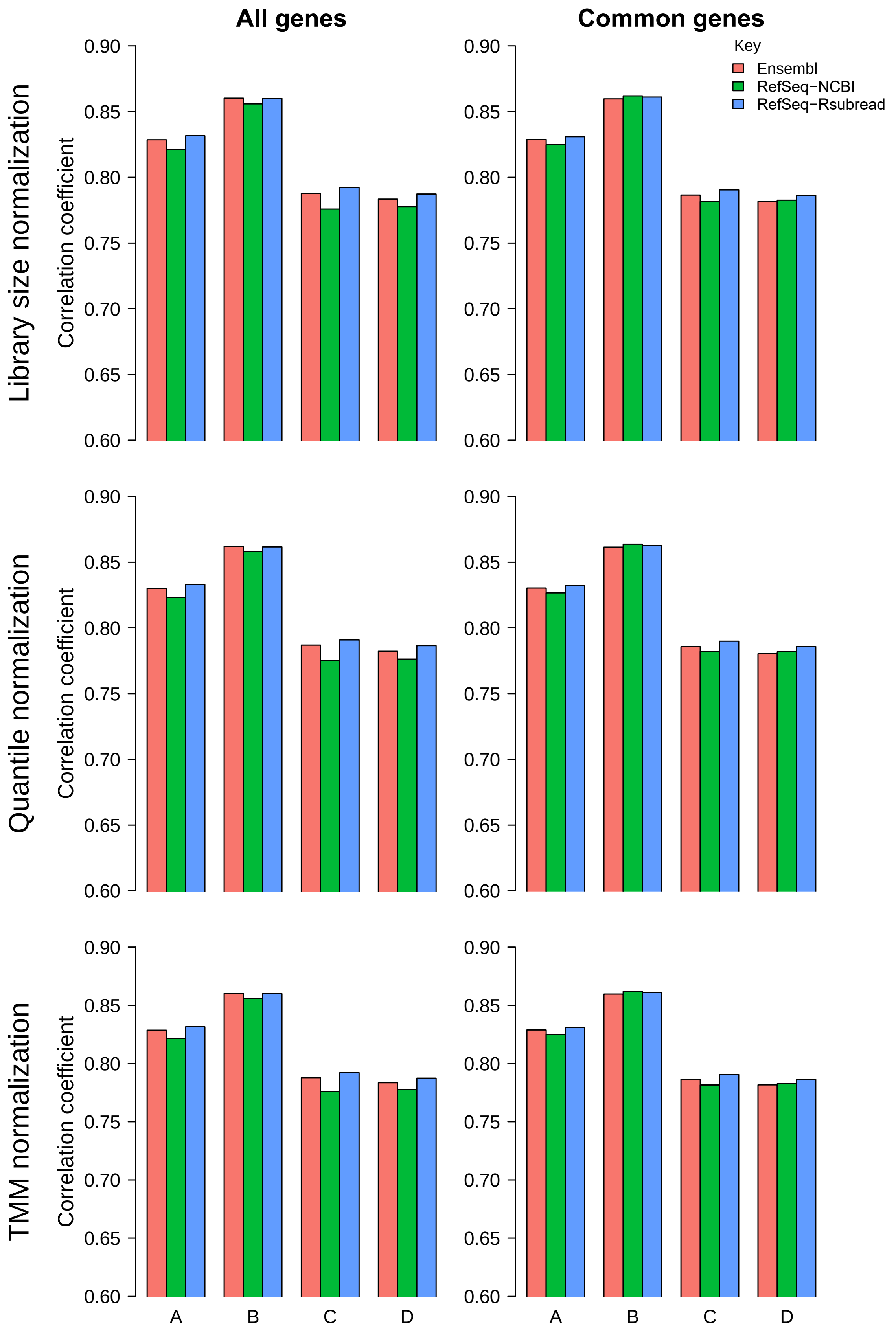

Figure S35. Pearson correlation analysis to compare RNA-seq data against TaqMan RT-PCR data. The analysis was performed based on the RSEM quantification results.

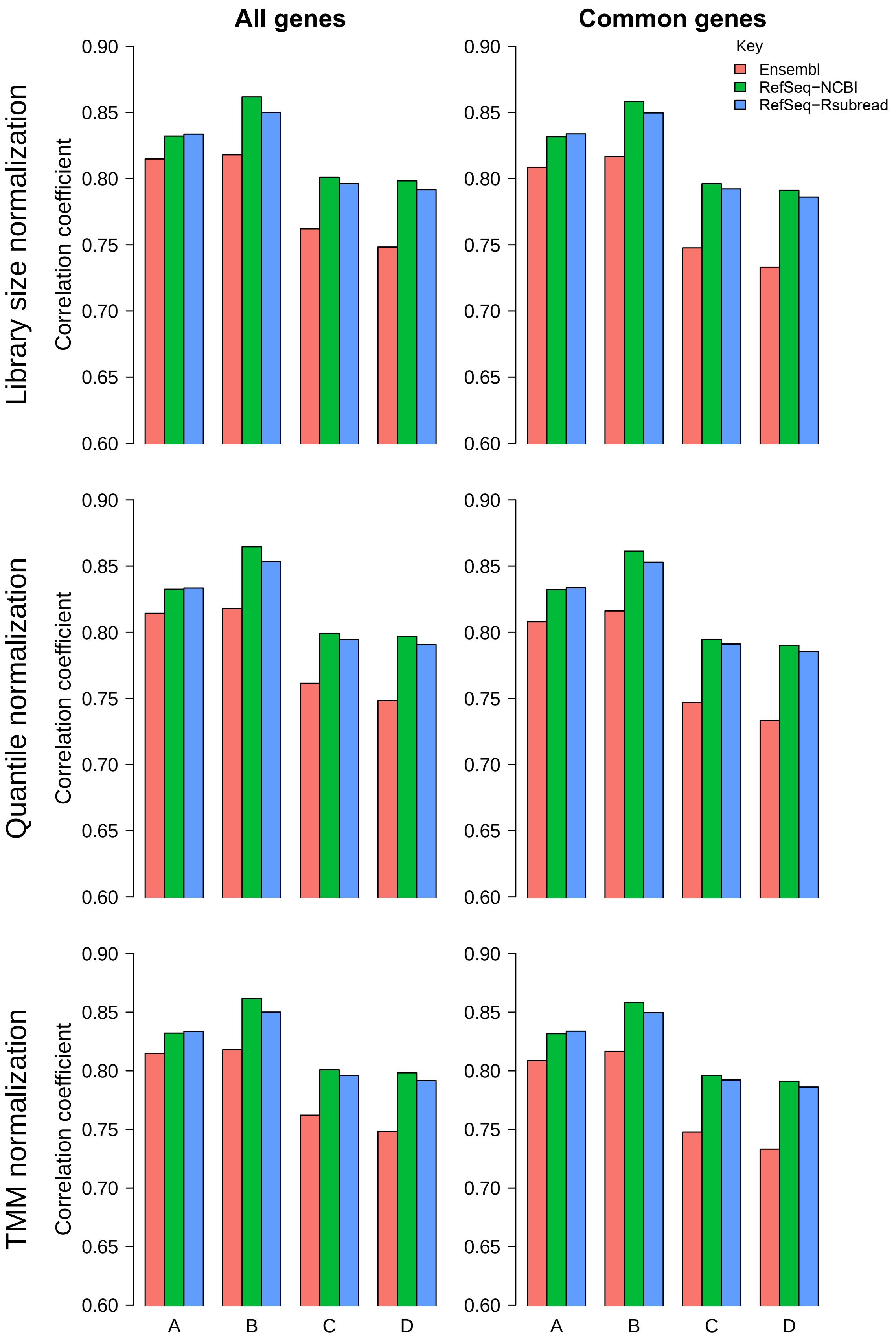

Figure S36. Pearson correlation analysis to compare RNA-seq data against TaqMan RT-PCR data. The analysis was performed based on the Kallisto quantification results.
